# *De novo* genome assembly of rice bean (*Vigna umbellata*) – A nominated nutritionally rich future crop reveals novel insights into flowering potential, habit, and palatability centric – traits for efficient domestication

**DOI:** 10.3389/fpls.2022.739654

**Published:** 2022-10-04

**Authors:** Tanushri Kaul, Murugesh Easwaran, Arulprakash Thangaraj, Arun Meyyazhagan, Mamta Nehra, Nitya Meenakshi Raman, Rachana Verma, Sonia Khan Sony, Khaled Fathy Abdel, Jyotsna Bharti, Gayacharan, Chandan Badapanda, Balamuralikrishnan Balasubramanian

**Affiliations:** ^1^Nutritional Improvement of Crops, International Centre for Genetic Engineering and Biotechnology, New Delhi, India; ^2^EuroEspes Biomedical Research Center, Institute of Medical Science and Genomic Medicine, La Coruña, Spain; ^3^ICAR - National Bureau of Plant Genetic Resources, New Delhi, India; ^4^Xcelris Labs Ltd., Ahmedabad, India; ^5^Department of Food Science and Biotechnology, College of Life Sciences, Sejong University, Seoul, South Korea

**Keywords:** *Vigna umbellata*, *de novo* assembly, draft genome, *Vigna radiata*, late flowering, palatable

## Abstract

Rice bean is a less-known underutilized legume crop with a high nutritional value among members of the Vigna family. As an initiative to compose rice bean (*Vigna umbellata*) genomic resource, the size of 414 mega-base pairs with an estimated identification of 31,276 high confidence index genes via 15,521 scaffolds generated from Illumina and PacBio platform 30X coverage data has achieved 96.08% functional coverage data from Illumina and PacBio platform. Rice bean genome assembly was found to be exquisitely close to *Vigna angularis* (experimental control/outgroup), *Vigna radiata*, and *Vigna unguiculata*, however, *Vigna angularis* being the closest. The assembled genome was further aligned with 31 leguminous plants (13 complete genomes and 18 partial genomes), by collinearity block mapping. Further, we predicted similar discriminant results by complete coding sequence (CDS) alignment. In contrast, 17 medically influential genomes from the National Institute of General Medical Sciences-National Institutes of Health NIGMS-NIH, when compared to rice bean assembly for LCB clusters, led to the identification of more than 18,000 genes from the entire selected medicinal genomes. Empirical construction of all genome comparisons revealed symplesiomorphic character in turn uncovering the lineage of genetic and functional features of rice beans. Significantly, we found deserving late-flowering genes, palatably indexed uncommon genes that regulate various metabolite pathways, related to abiotic and biotic stress pathways and those that are specific to photoperiod and disease resistance and so on. Therefore, the findings from this report address the genomic value of rice bean to be escalated *via* breeding by allied and applied approaches.

## Graphical Abstract

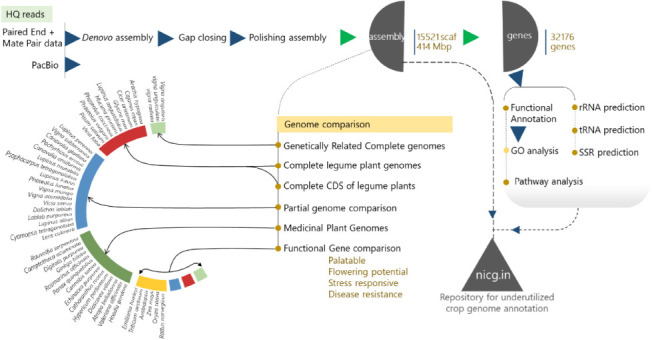

## Introduction

*Vigna umbellata* (Thumb.) is an orphan legume, multipurpose and lesser-known pulse with nutritionally rich crop. *Vigna* has been treated as an economically important genus that comprises of several domesticated species, including *Vigna radiata* (mung bean), *Vigna angularis* (adzuki bean), *Vigna unguiculata* (cowpea) (Dahipahle et al., 2019), and *Vigna mungo* (urad bean), etc. Intriguingly, Andersen et al. (2009) report that the rice bean VRB3 that is an underutilized crop has been nominated for multilocation testing among others and ranked no.1 with an average yield of 17.08 q/ha (Rana et al., 2014). However, the dis-adoption of rice bean by farmers is linked to the traits of landraces like late-flowering conditions [12-h light/dark photoperiod (Takahashi et al., 2015); 160 days post-anthesis (Joshi et al., 2007)] and palatable gestations to humans (Basu and Scholten, 2012). The underlying molecular biology of these conditions is unexplored and elusive. Consultative Group for International Agricultural Research (CGIAR), International Board for Plant Genetic Resources (IBPR), and International Centre for Underutilized Crops (ICUC) organizations form a network called Food Security through Rice bean Research in India and Nepal (FOSRIN), wherein rice bean has been labeled as one of the future crops foreseen for domestication by farmers in marginal lands (Basavaprabhu et al., 2013).

Despite the nutritional excellence of rice bean, the prevailing lack of awareness of its complete nutritional benefits means that rice bean can be categorized as an underutilized crop. Rice bean can become established and grow in various soil types. It is pest-resistant and presents tremendous potential as a nutritious fodder and high-quality grain. The major drawbacks of this pulse crop of the kharif season include late flowering, indeterminate nature, and tastelessness. Furthermore, there are meager possibilities for the improvement of rice bean due to its predominant landraces, nominal modern plant breeding, and limited seed supply. Consequently, the high diversity retained within its limited geographical distribution and the existence of few marketing channels mean that there is a great scope for the genetic improvement of rice bean. Despite the importance of rice bean as a multipurpose legume utilized for culinary purposes, the nutritious flour it produces, its use as a livestock feed, and its efficiency as an accumulator of nitrogen in soil, the exploitation of this species in terms of productivity is meager. There are several features of rice bean that require attention from geneticists before it can be widely adopted for standard consumption, including its high photoperiod sensitivity, late flowering period, high vegetative growth concerns, habit of twining (which makes harvest difficult), hard seededness, and seed shattering on maturity (Andersen, 2012). Furthermore, the vulnerability of rice bean to fungal pathogens results in heavy losses at harvest and reduced crop quality. The limited range of genomic tools for rice bean has impeded the increases in its yield over a given period of time until now. There is an urgent need for crop improvement and the domestication of rice bean to generate new varieties with high-yield traits as well as pleasant organoleptic properties and reduced levels of antinutrients (Gaur et al., 2018). Despite the fact that most of the available literature provides evidence of rice bean resistance to insect and aluminum toxicity (toxicity tolerance), no priority has thus far been given to understanding its flowering mechanism, palatability properties, and disease resistance profiles, which are the essential characteristics for achieving the maximum potential of this crop.

This study produced a detailed description of the nutritional attributes of this legume based on its genetic background. The results will increase an awareness of the nutritional excellence of rice bean and improve the genomic research on this crop. Our study involved the collection, screening, whole-genome sequencing, and genotype identification of rice bean starting from whole-genome sequencing with both the Illumina and PacBio platforms. A total of ∼69 Gb of data were generated from multiple sequencing platforms, including the Illumina (paired and mate pair) and PacBio platforms. Conventional genomic elements and novel genes for palatability domains, flowering potential, and disease resistance were identified through the whole-genome sequencing of *Vigna umbellata* (Thumb.) Himshakti in different accessions, landraces, and wild species to characterize genome-wide variations.

## Materials and methods

### Plant material

The known phenotype background of *Vigna umbellate* (Thumb.) VRB3 – indeterminate plant type with 8–10 seeds per pod, seeds are bold (7.56 g/100) and light green grain colored was provided by ICAR-NBPGR (National Bureau of Plant Genetic Resources), New Delhi for WGS and PRR-2010-2, PRR-2011-2, RBHP-307, RBHP-104, and EC-14075 for transcriptome study of selected genes for flowering, palatability, and stress response genes.

### DNA library preparation

A paired-end sequencing library was prepared using an Illumina TruSeq Nano DNA HT Library Preparation Kit (Kumar et al., 2017). A 100-ng sample of g-DNA for the 350-bp insert size (2 libraries) and a 200 ng sample of g-DNA for the 550-bp insert size were fragmented with a Covaris instrument according to the manufacturer’s instructions. The covariance program for the 350-bp insert size and 550-bp insert size was calculated.

A mate-pair sequencing library was prepared using the Illumina Nextera Mate-Pair Sample Preparation Kit (Campbell et al., 2016). 1 μg of the gDNA was simultaneously fragmented and tagged with a biotinylated mate-pair junction adapter. An AMPure bead purification step was performed to clean up the PCR product and remove the smallest fragments (<300 bp) from the final library. Quantity and quality checks (QCs) of the library were performed in a Bioanalyzer 2100 instrument (Agilent Technologies) using a high-sensitivity (HS) DNA chip.

### Comparative genome attributions

Genetic population structure analysis identified three distinct clusters in which the *Vigna* umbellata accession genome profiles and anchoring elements that correspond to functional features were mapped based on the sequences of *Vigna angularis* (PRJNA328963) from the Beijing University of Agriculture, *Vigna radiata* (PRJNA243847) from Seoul National University, and *Vigna unguiculata* (PRJNA381312) from the University of California, Riverside, which are genetically and functionally orthologous to *Vigna umbellate*. For all thirteen genomes selected for this study for the comparison of functional features, there are available complete genome profiles annotated *via* the eukaryotic genome annotation pipeline (EGAP). *Vigna angularis* was completely sequenced from 4 genome assemblies and 2 sequencing reads (Kang et al., 2015), and the total genome size was approximately 455 Mb, whereas the median total length of the *Vigna radiata* sequence (Kang et al., 2014) was 459 Mb, from 3 assemblies and 1 sequencing read, and that of *Vigna unguiculata* (Muñoz-Amatriaín et al., 2017) was 607 Mbp, from 2 genome assemblies.

### Gene predictions

Putative protein-coding genes were identified using the MAKER v2.31.9 pipeline (Campbell et al., 2014), which identifies repeats, aligns transcripts and proteins to a genome, produces *ab initio* gene predictions, and automatically synthesizes these data into gene annotations with evidence-based quality values. There were three steps followed to train the gene set: (i) For evidence collection and *ab initio* gene prediction, in the first step, repeat regions were masked internally using RepeatMasker (Tarailo-Graovac and Chen, 2009), which screens DNA sequences for interspersed repeats and low-complexity DNA sequences. Then, the sequences were checked for their likely associations with genes by aligning the transcripts (using Exonerate) and proteins (using NCBI BLAST) to the genome. In this case, we used the *Vigna radiata* protein and transcriptome dataset downloaded from NCBI (42284 proteins; GCF_000741045.1_*Vradiata*_ver6). The evidence was collected in GFF and FASTA format files. All these steps were carried out in: (i) the first round of MAKER execution to train the gene prediction software. (ii) In the second step, imperfect gene models from the first round of MAKER execution were used to the train gene predictor programs. Here, we used 2 gene prediction programs, Semi-HMM-based Nucleic Acid Parser (SNAP) (Korf, 2004) and AUGUSTUS (Keller et al., 2011). SNAP was trained using the MAKER bundled program, and AUGUSTUS was trained using BUSCOv3.0 (Waterhouse et al., 2017). The outputs from both programs were used as training sets for the final MAKER run (second round of MAKER execution) to predict genes. (iii) For final gene prediction as a final step, polished genes are predicted using transcript and protein alignments and the HMM models generated in step (ii). MAKER takes all the evidence, generates “hints” about where splice sites and protein-coding regions are located, and then passes these “hints” to the gene prediction programs.

### Genome library preparation

Genomic DNA was isolated from *Vigna umbellata* using an Xcelgen CP Plant DNA isolation kit, and libraries were prepared using an Illumina TruSeq Nano DNA HT library preparation kit for insert sizes of 350 and 550 bp. The mean sizes of the libraries were 664 and 438 bp for the 350-bp insert size and 876 bp for the 550-bp insert size. The library with an insert size of 350 bp was sequenced on the Illumina platform with 2 × 150 bp chemistry to generate ∼30 GB of data, whereas the library with an insert size of 550 bp was sequenced with 2 × 250 bp chemistry to generate ∼15 GB of data. The mate-pair library was prepared from a rice bean sample using an Illumina Nextera Mate-pair Sample Preparation Kit. The mean size of the library was 580 bp. The library was sequenced (2 × 150 bp chemistry) to generate ∼10 GB of mate-pair data. The Pac-Bio library was prepared and sequenced using the PacBio sequencing platform to generate 6.5 GB of data.

### Alignment and tree building

Geneious Prime (v2019.1) is used for the complete pre- and post-assembly annotation process. Paired reads were trimmed further using BBDuk (Kim and Daadi, 2019) in Geneious to remove low-quality bases (*Q* < 20) and discard reads shorter than 10 bp. The assembled reads were then used for *de novo* assembly to generate maps using the Geneious *de novo* assembler. The consensus sequences of the scaffolds responsible for flowering, palatability, disease resistance, and stress response genes were extracted from all selected species and aligned using the MAFFT (De Vega et al., 2015; Nakamura et al., 2018) plugin. Third-party plugins such as Mauve aligner (Darling et al., 2010), tfscan, TadPole, RepeatMasker, and the customized local search library, extended reassembly aligner, and maximum likelihood Geneious plugins were used. Domain trees were estimated using the maximum likelihood (ML) plugin.

The tRNAscan-SE tool v1.3.1 (Varshney et al., 2011) was used for the identification of selenocysteine tRNA-derived genes, repetitive elements, and pseudogenes from the assembled genome. A standalone version of tRNAscan-SE was used for the detection of tRNAs. Sequences encoding tRNAs were identified by tRNAScan-SE, which resulted in 577 tRNAs. Sequences encoding rRNAs were identified by RNAmmer (Zhuang et al., 2019), which resulted in 11 rRNAs.

Using the MISA program, highly polymorphic and ubiquitous SSR markers were identified from the assembled genome. The assembled genome from both samples was searched for SSRs using the MISA tool (Singh et al., 2012). A total of 67,523 SSRs were identified. Among the 67,523 SSRs identified, 55,699 exhibited a 150-bp flanking region that could be validated through PCR.

The bi-directional best hit (BBH) option was used to assign KO terms. Pathway analysis was carried out for assembled sequence using KAAS server (KEGG Automatic Annotation Server) by screening BBH. The genes were enriched in different functional pathway categories, predominantly related to metabolism, genetic information processing, environmental information processing, and cellular processes (Mochida et al., 2017). A total of 676 genes contributed to the activity of the carbohydrate metabolism pathway, with the next-largest group of 657 genes, were being related to signal transduction pathway.

Gene Ontology annotations were obtained for the annotated genes using BLAST2GO Command Line v-1.4.1. BLAST2GO PRO (Jain et al., 2013) that was used to annotate the functional genomic set for the assembled dataset. Gene Ontology terms fall under three categories: biological processes, molecular function, and cellular components. Some of the genes were assigned by more than one category. A total of 11,204 terms fell under biological process, 13,097 under molecular function, and 9,053 under cellular component.

To plot syntenic locally collinear blocks from Mauve alignment, the recently developed advanced real-time visualization tool Circa was used. Both the unidimensionality and connectivity of the two genomic datasets were plotted with Circa (OMGenomics, 2019).

### Mauve aligner

Multiple copies of the gene library were generated and aligned for comparison with all three reference genomes (Cheon et al., 2019). The consistency or variation among the reference genomes was determined to compute locally colinear blocks.

PlasMapper generates plasmid maps along with annotations. It provides multiple predictions, such as replication origin, promotor, terminator, selectable marker, regulatory sequence affinity tag, miscellaneous feature, report gene, and restriction site predictions (Shi et al., 2017). The *Vigna umbellate* draft assembly was subjected to identify these elements to report additional genome-editing scaled tools.

### Primer designing and specificity check

To check the expression levels of different genes, primers were designed employing Primer 3 online software available in NCBI, which was followed by specificity check against *Vigna angularis* database using BLAST software. The gene names and primer sequences are tabulated in Supplementary Excel 1.

### RNA isolation and synthesis of first-strand cDNA

Leaf samples from rice bean (VRB-3) plants (25 days post-germination) were used for total RNA extraction employing Tri-Reagent (Sigma), according to the manufacturer’s protocol. To avoid genomic DNA residue, Dnase treatment was executed by adding 1 μl of Dnase solution (8 μl of 10X Dnase buffer, 3U Dnase mixed in H_2_O) to 10 μl of each RNA sample followed by incubation at 37°C for 10 min. The RNA sample treated by Dnase was further used for the synthesis of first-strand cDNA utilizing Superscript III reverse transcriptase kit (Agilent Technologies) and oligo (dT) primer in a total volume of 20 μl, according to user manual instructions.

### Validation of the predicated genes by quantitative real-time polymerase chain reaction

The diluted cDNA was used as a template for quantitative real-time polymerase chain reaction (qRT-PCR) and amplified by gene-specific primers (Table no) using SYBR Green PCR Master mix (Agilent) on Real-Time PCR (Agilent Technologies, United States) system. To set up the RT-PCR, the following conditions were employed; 95°C for 5 min, followed by 40 cycles each of 95°C for 10 s, 60°C for 15 s, and 72°C for 15 s. qRT-PCR was performed with three technical replicates along with NTC. Expression of 2-oxoisovalerate dehydrogenase gene in each sample was used to normalize gene expression values. The standard error and standard deviation were calculated from replicates, and significance was measured at the level of *p* ≤ 0.05.

## Results

### *De novo* assembly and genome statistics

**FIGURE 1:**
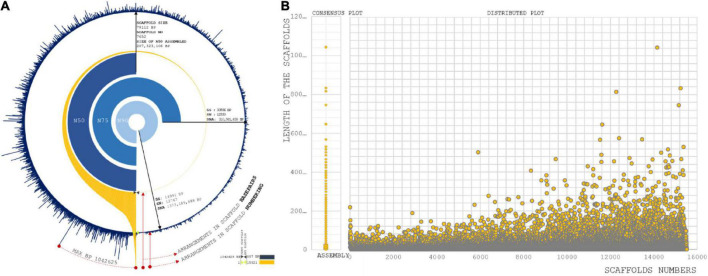
**(A)** Assembly summation statistics: First layer (Blue) exhibit the distribution of scaffold number orderly over completeness of 15,221 scaffold reads. Second layer (Orange) are the arrangement of scaffolds with respect to base pair length. Internal three layers are representing N50, N75, and N90 statistic of our assembly with scaffold size, scaffold position, and size of summation statistics. **(B)** Sequence assembly length distribution coordinated from scaffold numbers to length of the sequence in each scaffold.

With the raw data of 69.32 GB, 414 Mbps draft size were assembled using the Allpath LG (APLG) assembly pipeline (Kang et al., 2015). A total of 17,601 scaffolds were produced prior to gap closure and post-polishing with 3 GB of PacBio data. We obtained 15,521 final scaffolds and 36,461 contigs. A total of 41,447 and 39,387 contigs were reported during the process of gap closure using PGJelly for high confidence draft assembly (Peng et al., 2019). The length of the total assembled reads was 387,954,823 bp; post-improvisation, a total of 414,706,990 bp was obtained from Pbjelly, which aligns long sequencing reads. The average scaffold size was 26,719 from the final assembled reads (Supplementary Table 1). The N50, N75, and N90 metrics were applied to weigh the quality of the genome assembly (Figure 1A). The size of the N50 distribution is 207,323,106 bp. The scaffold size distribution and summation statistics are mentioned in Supplementary Table 2.

### Length distribution

#### Assembly

A total of 779 scaffolds were between >8,000 and ≤10,000 (Supplementary Table 3). Significantly, more than 7,343 assembled scaffolds were greater than 10,000 bp in length, which increases the possibility of understanding gene conservation and diversity in comparison with closely and distantly related species (VanBuren et al., 2015; Figure 1B). *Genes predicted:* A total of 31,276 genes were predicted from 15,521 scaffolds. The maximum length of the genes involved in the prediction analysis was 15,628 bp. A total of 8,344 genes were distributed with the higher ranges of >8,000 and ≤10,000, >6,000 and ≤8,000, >4,000 and ≤6,000, and >2,000 and ≤4,000 (Supplementary Table 4). A total of fourteen genes were predicted to present a size >10,000.

### Comparative genome statistics

**FIGURE 2:**
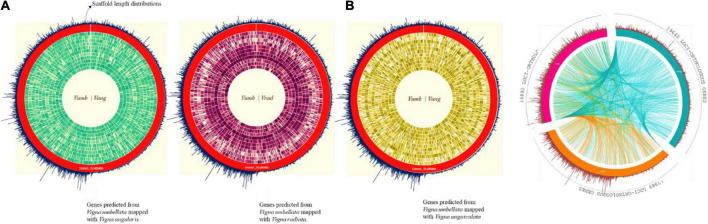
**(A)** Complete predicted genes *Vigna umbellata* mapped for Chromosomal-specific comparison for the proximity of gene scaffold mapping to its reference. (i) Rice bean mapped syntenic region average of 82% spanned for its gene specificity with adzuki bean. (ii) Rice bean with mung bean having relation of each chromosomes of about 72%. (iii) Rice with cowpea mapping found 62% of functional clusters with micro break from its evolution. **(B)** Orthologous synteny gene map comparison between adzuki bean, mung bean, and cowpea with genes mapped from assembly. Green color is adzuki bean mapping with 19,640 loci, orange colored mung bean mapping; 17,989 loci, and 16,892 loci for cowpea-pink colored.

About 80% of the functional annotation of genes from the assembled scaffolds were annotated from *Vigna angularis* (Tomooka, 2009), the species which is closely related to *Vigna radiata* and *Vigna unguiculata genotypically* (Figure 2A). For reported *Vigna angularis* genome, 443 Mbps were assembled from 290 GB of raw data, whereas for rice bean, 15,521 scaffolds were assembled from 414 Mbps (Kang et al., 2015) which is about 30X- coverage of raw read library sequence (Supplementary Table 5). The total numbers of scaffolds from mung bean and adzuki bean were 2,748 and 3,883, respectively, and for rice bean, 15,521 scaffolds predicted 31,276 genes.

### Alignment read distribution for comparative genome annotations

The total length of the predicted genes was 49 Mbp from 414 Mbp of assembled reads. Rice bean predicted genes were mapped with 3 reference species; adzuki bean –105,415,055 bp, mung bean – 92,715,800 bp, and cowpea – 81,760,762 bp (Supplementary Table 6). A total of 41,525, 32,657, and 16,614 reads were aligned to adzuki bean, mung bean, and cowpea, which led to find 21,006, 21,657, and 20,664 genes, respectively (Supplementary Table 7).

### Gene profile and protein-coding regions

Orthologous loci were found for 16,892 out of 31,276 genes (Figure 2B) by mapping to adzuki bean (Supplementary Table 8). We found 23,490 loci of coding sequence (CDS) regions in adzuki bean and 16 Mbp of non-coding RNA region by mapping to its reference genome (complete LOC IDs are provided in Supplementary Excel 2).

### tRNA operons

**FIGURE 3:**
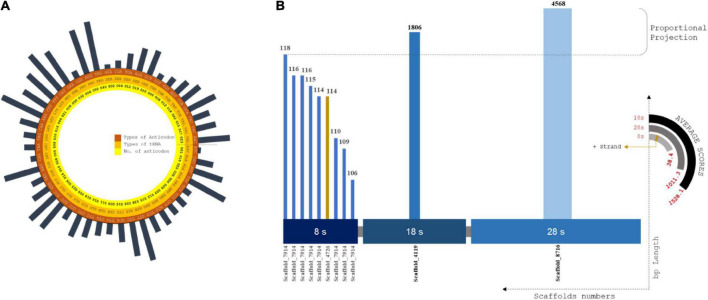
**(A)** Circular plot of tRNA composition from scaffolds assembled: Layers of tRNA distributed as codons, amino acids and no. of codons screened. Largest codon usage by Proline (TGG) which is of 93 nos. and the lowest codon usage is Aspartame, i.e., 1 of ATC codons. **(B)** rRNA plot by distributed size of ribosomal RNA subunits against the scaffold utilized for prediction using RNAmmer. X coordinates are subunit classification and y coordinate classifies scaffold count and length of each scaffolds. The pseudo-semi-circular projections depicts the score value of each prediction and strand profile of predicted rRNA profiles.

Codon usage biases and the use of identical anti-codons for tRNA pseudogenes are caused by retrotransposon-driven repetitive elements (Gibbs et al., 2004). A total of 577 tRNA operons screened that were positioned in intronic spacers and bound regions. The lowest covariance model score (Inf Score) calculated was 20.3, and the highest was 92.3. Arg-Leu-Ser showed the maximum frequency among the predicted codons (Supplementary Table 9). The total tRNA genes are summarized in the Supplementary Excel 3 (Figure 3A).

A total of 34 tRNA bound regions were found in intronic spacers, with internal scores between 92.4 and 84.2. RNA self-splicing is predetermined by these regions, which tends to translate the number of amino acids from the predicted genes (Smathers and Robart, 2019). A total of 577 tRNA loci (excluding 34 pseudo-tRNAs) from 543 scaffolds, spanning 430 bp with an average length of 20 bp, were predicted (Supplementary Table 10).

Interestingly, only tRNA genes encoding Tyr, Met, Lys, Asn, Ser, Ala, and Leu showed the presence of introns. The predicted tRNA genes indicated the maximum density of copies on the scale of the assembly contribution.

### rRNA cluster fragment distribution

The alignment for rRNA prediction was placed farther downstream to increase the good number of model scores. A total of twelve full-length tandem arrays of rRNA genes were found in the assembled scaffolds (Figure 3B). In total, 4 scaffolds were involved in the rRNA prediction calculation, and we observed an average length of 672 bp. The highest match of rRNA is 4568 bp. Then, 8S, 18S, and 28S rRNA units were predicted to have lengths of 113, 1,806, and 4,586 bp, respectively. One positive strand was related to 8S rRNA genes of approximately 114 bp length (Supplementary Table 11). No positional overlap was found between the predicted tRNA and rRNA scaffold regions.

### SSR marker analysis: Compound repeat annotations – Class I

**FIGURE 4:**
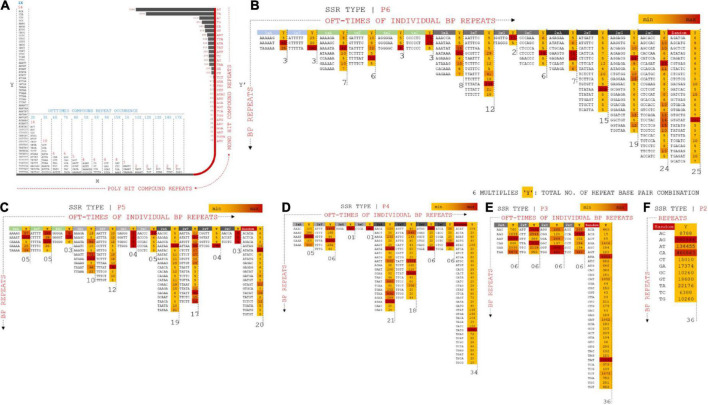
**(A)** Compound repeats from assembled reads were plotted from the distribution of offtimes of repeat occurrence and number of hit (mono and poly hits). X-Y coordinate prevails the distribution of compound repeats in multiple combinations whereas X–Y’ projects mono repeats with multiple form bp combinations. **(B)** P6 SSR operons mapped for distributed combinations for 5xX (A, T, G, C), 4xX, 3xX, 2xX, and random specificity of repeats. Operons are projected on positioning oft-time count, Y multiples of each marker types. **(C)** P5 SSR operons mapped for distributed combinations for 4xX (A, T, G, C), 3xX, 2xX and random specificity of repeats. **(D)** P4 SSR operons mapped for distributed combinations for 3xX (A, T, G, C), 2xX and random specificity of repeats. **(E)** P3 SSR operons mapped for distributed combinations for 2xX (A, T, G, C) and random specificity of repeats. **(F)** P2 SSR operons mapped for distributed combinations for random specificity of repeats.

Nucleotide diversity analysis of *Vigna umbellata* draft assembly provided a rich resource of polymorphic SSRs, which are useful to determine marker-trait relationships, especially for quantitative trait loci and molecular breeding. Repeated operons for hundreds of polymorphic non-monomeric SSRs and flanking bases from the assembled data were cataloged to translate them into genetic markers (Vining et al., 2017). The repeats were categorized based on the position and number of repeats of each allele. The compound repeats (C_r_) were calculated for stable (c) and variable (c*) alleles (Figure 4 and Supplementary Excel 4). C_r_ presented a maximum scaffold length of 9,711 bp for the stable allele (SA), whereas the variable allele (VA) presented a length of 1,048 bp. The length of the repeat loci was 71 bp on average (median statistics) for SA and 92 bp for VA. The number of C_r_ poly-hits ranged from 2x–54x, whereas the 32-single ranged from 19 to 5284 combination repeats. A total of 2,111 (0.1%) six-nucleotide repeats in assembled reads, 2,045 (0.1%) P5 repeats, 29,524 (1.8%), 76,493 (6%) 3-bp repeats were found in the distribution of non-compound SSR marker screening.

### BLASTx analysis

**FIGURE 5:**
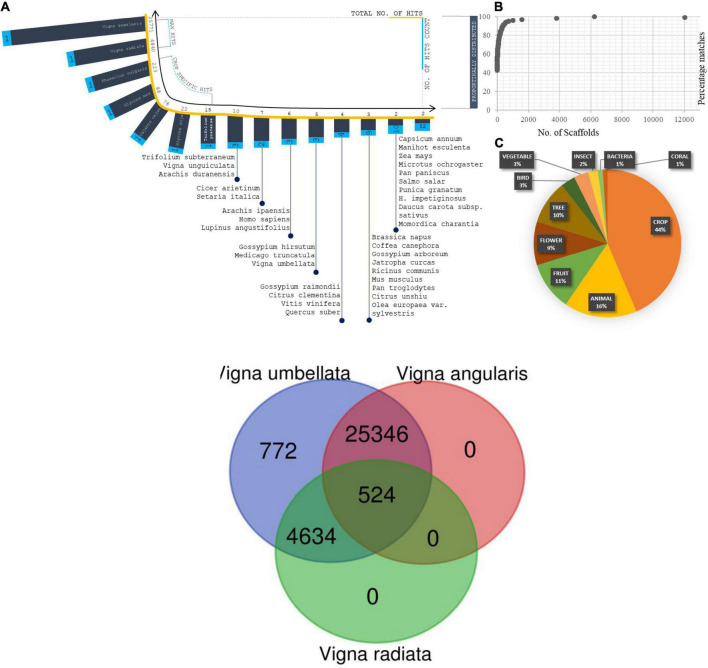
**(A)** Distribution of Species from total scaffolds matched from blastX Analysis. Coordinates projected in terms of scaffold matches (total no. of hits and no. of hits counts). **(B)** Percentage distribution of scaffolds in correspondence of species fall. **(C)** Plot on total organism matches with its generic names. **(D)** Enzyme composition predicted from scaffolds and projected the plot based on enzyme classification and enzyme reactions types. Two coordinates corresponds the scaffold distribution and no. of enzyme reaction types. Totally five enzyme classifications were analysed and plotted against reaction types and scaffold distributions.

**FIGURE 6:**
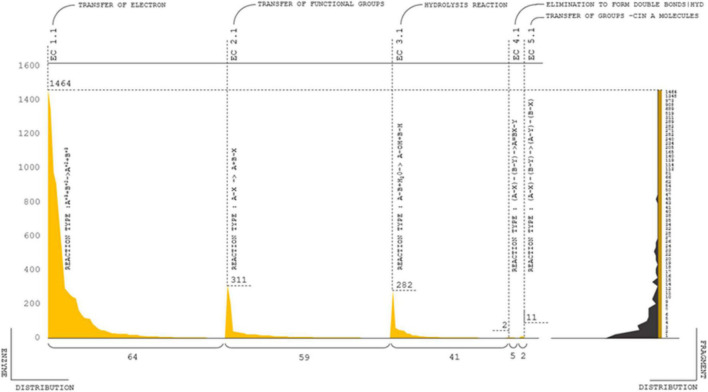
Annotated Genes of *Vigna umbellate* shared between *Vigna angularis* and *Vigna radiata*.

All predicted genes were annotated by evaluating their homology through BLASTX searches against the NR database (non-redundant database) for complete integration with the physical/genetic maps (Zhao et al., 2019). The genes were aligned using the following optimized search parameters: gap opening penalty (G) = 4, gap extension penalty (E) = 1, mismatch score (q) = -1, match score (r) = 1, word size (W) = 11, and *e*-value < e-20 (Singh et al., 2004). The total number of genes predicted from the assembly was 31,276, including 31,237 genes with BLAST hits and 39 genes with no BLAST hits. The average gene length was 1.8 kb, and the average GC content was 42%. A total of 7,708 scaffolds were associated with enzymes: 279 were membrane-specific; 1,418 were related to the chloroplast response; 759 were related to the mitochondrion response; 442 were ribosome-specific; 1,082 were transcription related; 89 were related to leucine-rich-repeat regions, and 69 were translational-related. *Scaffold percentage distributions:* The top species hits ranked 1 for *Vigna angularis*, second with *Vigna radiata* and *Vigna unguiculata* as third closer genome, which were calculated from the assembled reads (Figure 5). The total% distribution ranged from 100 to 43. Major crops such as *Glycine max*, *Glycine soja*, *Zea mays*, *Vigna aconitifolia*, and the *Oryza sativa* japonica group presented throughout the alignment frequency. A total of 90% of the crops from the 100% index fell within the 99–97% index matches. The counts of scaffolds are proposed to be used to study the number of scaffolds (Figures 6A–C). A total of 6,235 scaffolds came from the 100% distributions, 2,139 scaffolds from 99 to 90%, 2,297 scaffolds from 89 to 80%, 971 scaffolds from 79 to 70%, 284 scaffolds from 69 to 60%, 47 scaffolds from 59 to 51%, and 10 scaffolds from 49 to 43%. Divergence plots are classified based on the crops and maximum hits. A total of 25,771 scaffolds came from *Vigna angularis*, 4,840 from *Vigna radiata*, 223 from *Phaseolus vulgaris*, 88 from *Glycine max*, 78 from *Cajanus cajan*, 22 from *Glycine soja*, 15 from *Trifolium pratense*, and 22 from other species.

### Gene ontology analysis of rice bean draft genome sequence

Functional descriptions were assigned to predict rice bean genes. Genes associated with similar functions are assigned to a similar GO functional group (Xu et al., 2017). This study identified several potential genes involved in response to palatable indexed genes, flowering potential, stress response, and disease resistance genes to various metabolic processes, and the regulation of transcription and translation. The total number of GO-annotated genes was 16,974, among which the overlapping positional map included 11,204 biological processes, 13,097 molecular functions, and 9,053 cellular components predicted from the genes. These findings should help to identify and manipulate rice genomic diversity to contribute to the development of crops.

#### Enzyme functions

The GO-profiled enzymes were classified into seven categories: oxidoreductases, transferases, hydrolases, lyases, isomerases, and ligases, which varied depending on the responses that the enzymes catalyzed (Morgat et al., 2020). The most common types of enzymes were oxidoreductases, transferases, and hydrolases. Distinct enzyme classes were further systematically categorized based on the substrate reaction and mechanism of response. The composite enzyme contributions were categorized as E.C 1-2-3-4-5, whereas there were a total of 184 top-level classifications and 10,531 complete classifications (Figure 6D). The highest distributions were accompanied by approximately 1,464 major | 64 subclassified E.C 1.1 reaction types, e.g., electron transfer:A^+3^ + B^+2^- > A^+2^ + B^+3^. Following the electron transfer mechanism, 311 | 58 enzymes were classified as E.C 2.1; state of functions derived from the transfer of functional groups; the reaction type is A–X – > A + B-X. The total number of enzymes from the 3.1 nomenclature was 282 | 41 predicted; the reaction type was hydrolysis [A-B + H_2_ 0 – > A- OH + B – H]. E.C 4.1 and 5.1 are 1 | 5 and 11 | 2 combinations of reaction types, respectively (Supplementary Table 12); i.e., elimination to form double bonds [(A-X)-(B-Y)- > A = BX-Y] and transfer of groups within molecules [(A-X)-(B-Y)- > (A-Y)-(B-X)]. The complete, curated enzyme classifications are provided in the Supplementary Excel 5. Other molecular functions are about 292 | 5,741 genes major and subclassified functions in the cellular mechanism category (Supplementary Table 12).

#### Biological process

A total of 166,622 independent annotations with 23,907 dependent genes were assigned GO terms, among which the genes predicted from the assembly exhibited 11,204 functions related to biological processes. The 6,957 prevalent functions corresponding to biological processes from rice bean were related to ion-dependent functions, with predominance of membrane-transporter activity and hemostasis. The second largest category among the genes associated with DNA dependence included approximately 1,560 functions involved in replication, transcription, translation, endonucleolysis, repair mechanisms, topological changes, duplex unwinding, and signal transduction. Approximately 918 identified membrane functions play critical roles in transport mechanisms, such as ion, electron, antigen, amino acid, and ATP transport, as well as signaling and response mechanisms.

### Kyoto encyclopedia of genes and genomes ortholog assessment

A total of 515 functional orthologs were derived from 19,173 experimentally characterized Kyoto Encyclopedia of Genes and Genomes (KEGG) gene-associated pathways. The pathway annotations included the functional hierarchies and binary relationships of biological entities.

#### Gene distribution in Kyoto encyclopedia of genes and genomes annotation pairs

**FIGURE 7:**
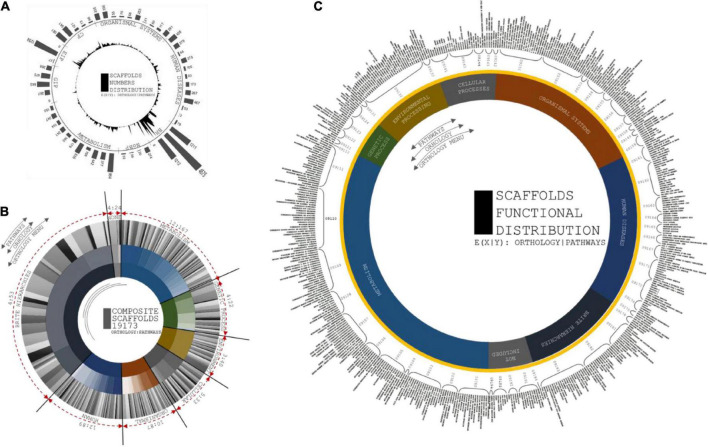
**(A)** Circular and distribution peaks are projected from number of scaffolds involved in each orthologs. First layer of circular projections shows the maximum peaks of scaffolds distributions from each pathways categorized in orthologs. Second layer depicts the number of pathways with respect to scaffolds involvement to predict its pathway functions. **(B)** Circular distributions of pathways from ortholog head. Composition of three layers are coordinated by number orthologs, number of pathways from each orthologs, and scaffolds distributions at each pathways. First layer projected for number of orthologs from KAAS server, second layer depicts the number of pathways from seven ortholog head, and third layer distributed based on scaffold involved in each pathways predicted. **(C)** Circular projections of functional specification of Pathways predicted from KASS automated server. Plot coordinates are differentiated in three layers. First layer specifies orthology menu of the pathways from prediction profile. Second layer specifies compositional head of pathway ID and third layer is to specific functions of each pathways that corresponds from orthologs.

A total of 54 functional hierarchies were predominantly related to the correspondence of orthology with KO identifiers (Ho et al., 2018). A total of twelve metabolic hierarchies included 167 orthologs. A total of four genetic information processing hierarchies exhibited 22 orthologs. A total of three environmental information processing hierarchies exhibited 40 orthologs. A total of five cellular process hierarchies exhibited 33 orthologs. A total of ten organismal system hierarchies exhibited 87 orthologs. A total of 4 BRITE hierarchies exhibited 53 orthologs (Figure 7). The significance of the composite gene distributions for the hierarchies demonstrated functional consistency in the metabolic resources and disease-specific mechanisms. Triangulation of multiple vertices of pathway annotations empirically contributes to the expectancy of obtaining rice bean genome annotations.

Kyoto Encyclopedia of Genes and Genomes analysis showed that most of the expanded genes were involved in signal transduction, protein metabolism, ribosomes, DNA repair mechanisms, and mitochondrial biogenesis, clearly showing that the rice bean genomes represent a huge repository of resources for domesticating crops according to the functional classification of the rice bean genomes. The expanded orthogroups were involved in phenylpropanoid, terpenoid, carotenoid, tropane, piperidine, flavonoid, folate, zeatin, isoquinoline, cutin, sesquiterpenoid, stilbenoid, gingerol, prodigiosin, monoterpenoid, phenazine, novobiocin, isoflavanoid, indole, acridone, benzoxazinoid, clavulanic acid, puromycin, and staurosporine biosynthesis (Kanehisa and Sato, 2020). We observed that 515 genes from contracted gene families clustered in 54 KEGG pathways, including starch, sucrose, purine, pyruvate, inositol, glutathione, glyoxylate, and pyrimidine metabolism (Supplementary Table 4). The patterns of the genes encoding enzymes distributed in the metabolite pathways indicated the enrichment of hierarchies associated with flavonoid biosynthesis.

### Transcription factors

Plants regulate intrinsic gene expression by transcription factors (TFs), transcriptional regulators (TRs), chromatin regulators (CRs), and the basal transcription machinery (Dai et al., 2013). Recruitment of TF-binding domains from transposases or integrases is a recurrent theme in evolution (Chen et al., 2016; Borrill et al., 2019). In total, 11,202 TFs and 15 gene clusters of transcription factors with extensive functions were studied in rice bean genome. (Supplementary Table 13), 38% of the total functional homolog count of these genes were identified in comparison with adzuki bean mapping, and 61.51% (293,575 locus) of Hexamer motif 5′-ACGTCA-3 (transcription factor HBP-1a) found in rice bean genes, which promotes histone gene binding (Cao et al., 2019). 36 of 14.54% (69,389 locus) of nitrilase family member 2 (NIT2) identified, which cleaves carbon-nitrogen bonds (Urbancsok et al., 2018). Less than 10% of other TFs, such as RNA1-encoded 60-kDa nucleotide-binding proteins, are usually involved in the hybrid systems of plant proteins (Carette et al., 2002), opaque endosperm 2 (opaque-2), which regulates the expression of many members of the zein multigene family of storage proteins (Krishna et al., 2017, SEF4) nuclear factor (Zhao et al., 2015), common plant regulatory factor-promoting genes (CPRF1), which are regulated by diverse stimuli, such as light induction or hormone control (Rügner et al., 2001). TEA/ATTS transcription factor [abaA] (Hwang et al., 1993), putative chromosomal passenger protein (CPC1) (Carmena et al., 2012), G-box-binding factor (GBF) (De Vetten and Ferl, 1995), facilitative glucose transporter (GT-1), facilitative glucose transporter GT2 (GT-2) (George Thompson et al., 2015), sequence-specific single-strand DNA-binding protein-2 (ssDBP-2) (Takase et al., 1991), TATA-box-binding protein-associated factor 1 (TAF-1) (Friedrich et al., 2005), and putative homeodomain-like transcription factor superfamily protein (HOX1a) (Comelli et al., 1999). Expansion and contraction of TF gene families might influence the regulation of biological functions and trait differences in rice bean (Joshi et al., 2016).

### Genome editing tools *Vigna umbellata* assembly

Editing tools have the potential to precisely change the architecture of a genome with the desired precision. These tools can modify the genomic architecture precisely to achieve the required accuracy (Zhang et al., 2018). These tools have been efficiently used for trait discovery and for the generation of crops with high crop yields (Wang et al., 2019). This analysis enables the investigation of evolutionary elements and the molecular functional impact of domestication and breeding *Vigna umbellata*. A total of three important genetically close traits must be addressed during the domestication process maker rice bean a viable crop: palatability, latency, and an increased rate of flowering (Pattanayak et al., 2019). A total of nine major functional clusters were derived from 31,276 predicted genes with several loci and counts are listed in the Supplementary Table 14 (Dong et al., 2004); i.e., vectors that exhibit plasmid specificity, hybridization loci, localization sequences, ORI regions, promoter sequences, reporters, Tags, and terminator sequences.

### Genome map distributions

Gene mapping was performed for 414 Mb of the assembly, and 31,276 genes that were conserved in diverged plant species were found in the database. **[a]** A total of 49 Mb of genes predicted from the alignment of adzuki bean (455 mb), mung bean (459 mb), and cowpea (607 mb); **[b]**105 Mb of genes mapped from adzuki bean genome*;* **[c]** 92 Mb from mung bean genome and **[d]** 81 Mb from mapping cowpea genome. Furthermore, to achieve the consistency of the genes deposited in the reference genomes, 49 Mb of predicted genes were aligned to the complete reference of *Vigna angularis*, and 105 Mb of genes were found, which was 2X times higher than in the predicted gene profiles. In total, 21,006 genes in rice bean were identified from adzuki bean genome.

### Synteny clusters

**FIGURE 8:**
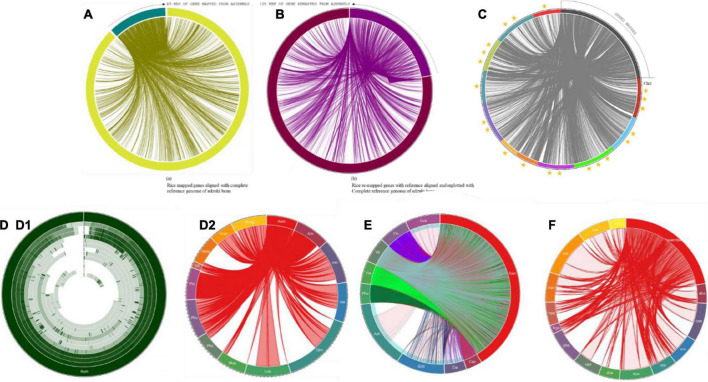
**(A)** Mapping of predicted genes with reference genome of *Vigna angularis*; gene distribution of 49 Mbp of genes with ref genome. **(B)** Mapping of genes predicted from remapping with reference genome of *Vigna angularis*; gene distribution of 105 Mbp of genes with ref genome. **(C)** Mapped genes rice bean are aligned with *Vigna angularis* chromosomes. Three star rated chromosome shares 40 Mbp of genome size. Two star rated shares >30 Mbp of genome size and single star rated chromosomes are 19 Mbp of genome map. **(D)** Circular distributions rice bean genome mapping with *Arachis hypogea*, *Cajanus cajan*, *Cicer arietinum*, *Glycine max*, *Lupinus angustifolius*, *Mucuna pruriens*, *Phaseolus coccineus*, *Phaseolus vulgaris*, *Pisum sativum*, and *Vicia faba*. **(D1)** circular distribution and **(D2)** Connective distributions of rice bean genome assembly. **(E)** Complete CDS coverage map of *Arachis hypogea*, *Cajanus cajan*, *Cicer arietinum*, *Glycine max*, *Lupinus angustifolius*, *Mucuna pruriens*, *Phaseolus coccineus*, *Phaseolus vulgaris*, *Pisum sativum*, and *Vicia faba* with rice bean assembled sequence. **(F)** Connective distributions of rice bean genome assembly with Medicinal genome, such as *Rauwolfia serpentine*, *Camptotheca acuminate*, *Digitalis purpurea*, *Ginkgo biloba*, *Rosmarinus officinalis*, *Panax quinquefolius*, *Cannabis sativa*, *Echinacea purpurea*, *Catharanthus roseus*, *Hypericum perforatum*, *Dioscorea villosa*, *Atropa belladonna*, *Valeriana officinalis*, and *Hoodia gordonii* species.

Extensive synteny among mapped and assembled reads allowed the integration of their genetic and physical maps. These observations support the hypothesis that detectable synteny exists between the maps and assembly related to divergence, although the conservation is more extensive than in the rice bean genome. The detectable synteny between these 105 and 49 Mbp of sequences provides clues for the identification of orthologous blocks associated with functional gene features with increased consistency. At a higher stringency, 80–90% of gene queries with seed weights were found among 653 identified homologs with an assembly that supported the quantification of the complete genome profile *Vigna umbellata* (Figures 8A,B).

Colinear blocks were calculated, and the flanking regions were plotted using Circa. The homology blocks between RmA (105 Mbp), RmM (92 Mbp), and RmC (81 Mbp) were closer, and high-degree comparisons identified the candidates for linkage and highly conserved profiles. The comparison of total genome positions revealed the coverage of >88% of the physical distance of the adzuki bean genome, with 42 conserved synteny blocks, 72% of the mung bean genome, and 62% of the cowpea genome, and many synteny microbreak points were found in all three selected reference species (Figure 2B). *Synteny map between rice bean and adzuki bean genomes:* The homolog richness of the *Vigna umbellata* genes mapped against entire 11 chromosomes of *Vigna angularis* was evaluated (Figure 2A). The alignment results revealed that Chr1, Chr3, and Chr9 were the chromosomes showing the high functional chromosomal alignment, for which the contributed genome size was greater than 40 Mbp. The conservation of synteny between Chr7, Chr2, Chr4, and Chr10 was closer to >30 Mbp, and Chr8, Chr5, and Chr11 constituted a group with low syntenic (micro) matches. The lowest conservation of homologs was found in chromosome 6, which corresponded to less than 19 Mbp of the total genome size (Figure 8C). The occurrence of prominent duplication events has been speculated because of the extent of collinearity blocks, which are more conserved and functional in relation *Vigna umbellata.*

### Comparison of 32 leguminous plant genomes with rice bean genomes

More than 14,000 species are reported as leguminous crops, including both edible and non-edible species (Ng, 2013). Leguminous annuals, perennials, shrubs, vines, and trees have adapted to a range of growing conditions around the world (Latef and Ahmad, 2015). Graham and Vance (2003) reported that thirty-two species are edible and are categorized as critically important economic food crops. Derived biological statistics from the FAO were used to list crops according to their economic significance based on the production range, post-anthesis period, and photoperiods of individual species (FAOSTAT, 2019). We selected 32 species that are edible, economically important, underutilized crops or staple crops for the comparison of the sequenced rice bean genome. The selected grains are intentionally grown for the harvesting of mature seeds, and these species are small fractional leguminous crops with lower production (Clement et al., 2019). Among the 32 species selected, the complete genomes have been reported for 10 species (6 chromosomal annotations + 4 raw annotations) (Supplementary Table 15), and for the other 21 species, 44% of ribosomal genes and other genes with various functions have been reported. The complete genome (Figure 8D) aligned with selected legume crops to understand the genetic makeup and versatility of the genome structure based on the genes predicted from rice bean assembly (Supplementary Table 16).

These crops were selected based on their photoperiod and post-anthesis period to improve multiparent advance generation inter cross-populations (MAGIC) for studying the genetic structure of rice bean traits and improving the breeding of rice bean accessions (Bandillo et al., 2013). The production range of these crops is influenced and fundamentally dependent by their flowering potential and photoperiod sensitivity. Rice bean requires a photoperiod with 12 h of daylight to flowering between 120 and160 days in a year (Joshi et al., 2007). The photoperiod range comprises much overlap compared with other species, and the species selected for this study is predicted to require a longer photoperiod than other crops but to present an equal nutritional potential. In the production range of rice bean with a prevalent 12-h photoperiod, 30,000 tons of production can be achieved in a year (Dhiman et al., 2010). According to Singh et al. (2004) rice bean is a crop with a smaller production range than other species requiring a 12-h photoperiod, such as *Vigna unguiculata*, *Mucuna pruriens*, *Vigna subterranea*, *Lens culinaris*, *Lupinus mutabilis*, *Vigna angularis*, *Phaseolus coccineus*, *Canavalia gladiata*, *Lupinus perennis*, *Phaseolus acutifolius*, and *Pisum sativum*. The production of these species ranges from 430,000 to 28,00,000 tons. The post-anthesis profile ranges from 70 to 150 days of the flowering period (Supplementary Table 17).

Phylogenetic evidence based on the ribosomal genes reported from selected species considering the rice bean data as an outgroup was used for the comparison of the genetic distances. (a) The scale range was calculated as 0.7 based on 55 nodes and 28 edge nodes. A distance of 0.01 was calculated for rice bean in the outgroup range in which *Vigna angularis* shared the closest monophyletic distance (0.02). (b) *Vigna angularis* and *Lupinus angustifolius* shared the same nodes located close to rice bean. The total length of the ribosomal RNA genes varied from 50to 133 bp.

Complete CDS were organized for alignment with the assembled rice bean sequence. A total of thirteen species, including *Vigna angularis*, *Vigna radiata* and *Vigna unguiculata*, *Mucuna pruriens*, *Phaseolus coccineus*, *Vicia faba*, and *Pisum sativum*, lacked a CDS annotation report. In total, 9 complete CDS sequences were aligned based on the LCB count, and the top 5 alignments shared more than 99% CDS alignment. The *Vigna angularis* CDS length covered approximately 99.8% of the total length, followed by that of *Vigna radiata* at 99.7%, *Vigna unguiculata* at 99.2%, *Phaseolus vulgaris* at 99.27%, and *Cajanus* at 99.2%, which were all aligned over more than 99% of the profile. *Cicer arietinum*, *Lupinus angustifolius*, *Glycine max*, and *Arachis hypogea* showed between 70 and 95% CDS alignment (Figure 8E).

The genomic data for the selected leguminous plants that for which there were not available complete genome annotations were downloaded (until July 2019 from NCBI FTP) and aligned with genes predicted from the rice bean assembly. The total number of sequences deposited from each species was subjected to BLAST searches against genes predicted from rice bean, and the total number of sequences that aligned over more than 300 bp was selected. A total of 61.54% (39 seq. out of 24 total seq) of the total *Pachyrhizus erosus* sequences were aligned with rice bean genes, and the corresponding values were 43.71% (789 seq. out of 1,805 total seq) for *Dolichos lablab*, 43.71% (789 seq. out of 1,805 total seq) for *Lablab purpureus*, 37.06% (391 seq. out of 1,055 total seq) for *Vigna mungo*, 34.13% (242 seq. out of 709 total seq) for *Lupinus luteus*, 29.36% (315 seq. out of 1,073 total seq) for *Vigna aconitifolia*, 26.64% (7017 seq. out of 26,343 total seq) for *Lens culinaris*, 19.34% (1908 seq. out of 9,864 total seq) for *Lupinus albus*, 19.25% (3190 seq. out of 16,569 total seq) for *Cyamopsis tetragonoloba*, 19.23% (10 seq. out of 52 total seq) for *Canavalia ensiformis*, 14.67% (11 seq. out of 75 total seq) for *Psophocarpus tetragonolobus*, 9.68% (3 seq. out of 31 total seq) for *Vigna subterranea*n, 8.32% (82 seq. out of 986 total seq) for *Phaseolus lunatus*, 7.89% (3 seq. out of 38 total seq) for *Canavalia gladiate*, 5.89% (67 seq. out of 1138 total seq) for *Vicia sativa*, 5.88% (4 seq. out of 68 total seq) for *Lupinus mutabilis*, and no sequence match with *Lupinus perennis* (Supplementary Table 18).

The complete genome sequences of selected species were subjected to the calculation of LCB with rice bean assembly. We found that *Vigna angularis*, *Vigna radiata*, and *Vigna unguiculata* presented the closest genetic profiles based on the annotation of the assembly with the corresponding genomes. The resultant alignment percentages were 88.29% (total LCB aligned 512 Mbp sequence of 521 Mbp) for *Phaseolus vulgaris*, 86.56% (523 Mbp of 592 Mbp) for *Cajanus cajan*, 80.55% (64 Mbp of 80 Mbp) for *Vicia faba*, 79.06% (74 Mbp of 979 Mbp) for *Glycine max*, 63% (530 Mbp of 366 Mbp) for *Cicer arietinum*, 53% (360 Mbp of 609 Mbp) for *Lupinus angustifolius*, 25.25% (93 Mbp of 371 Mbp) for *Phaseolus coccineus*, and 14.57% (57 Mbp of 397 Mbp) for *Mucuna pruriens*.

### Genome alignment of 14 metabolically and pharmaceutically active medicinal plant genomes

A total of fourteen taxonomically diverse and pharmaceutically important medicinal plant genomes reported by the Medicinal Plant Genomics Resource [MPGR] (2019) Michigan State University, United States, were aligned with the rice bean genome to identify an unparalleled resource for human health. For *Rauwolfia serpentine*, the greatest linear block of the medicinal genome aligned with rice bean corresponded to approximately 43.35% (161 Mbp) (Figure 8F) of the total rice bean sequence alignment and 89.63% (179 Mbp out of 414 Mbp) of the total genome size of *Rauwolfia serpentine*. For the other medicinal plant genomes, the alignment percentages are listed in Supplementary Table 19. Major metabolically functional enzymes to produce medicinally significant metabolites identified from rice bean gene mapping with selected medicinal plants, such as putrescine-N-methyltransferase, hyoscyamine 6 beta-hydroxylase, strictosidine synthase, 1-deoxy-D-xylulose 5-phosphate reductoisomerase, olivetol synthase, (+)-alpha-pinene synthase, (-)-limonene synthase, cannabidiolic acid synthase, secologanin synthase, tabersonine 16-hydroxyla—, NADPH–cytochrome P450 reductase, desacetoxyvindoline 4-hydroxylase, loganic acid methyltransferase, 3-beta hydroxysteroid dehydrogenase, phenolic oxidative coupling protein, octaketide synthase, perakine reductase, Acetyl Ajmalan Acetyl Esterase, polyneuridine aldehyde esterase, raucaffricine-O-beta-D-glucosidase, vinorine synthase, and arbutin synthase. These enzymes are responsible to produce clinically important ligands, i.e., carnosic acid, rosmarinic acid, oleanolic acid, acevaltrate, hesperidin, ajmalicine, rescinnamine, serpentine, ginsenosides Rb1, hypericin, amentoflavone, hoodigogenin A, gordonoside F, ginkgolide, bilobalide, cichoric acid, echinacoside, prosapogenin A, gitoxigenin, vincristine, tabersonine, secologanin, cannabidiol (CBD), 9-methoxycamptothecin, secologanin, and atropine.

### Gene pooling for rice bean domestication and crop improvement

**FIGURE 9:**
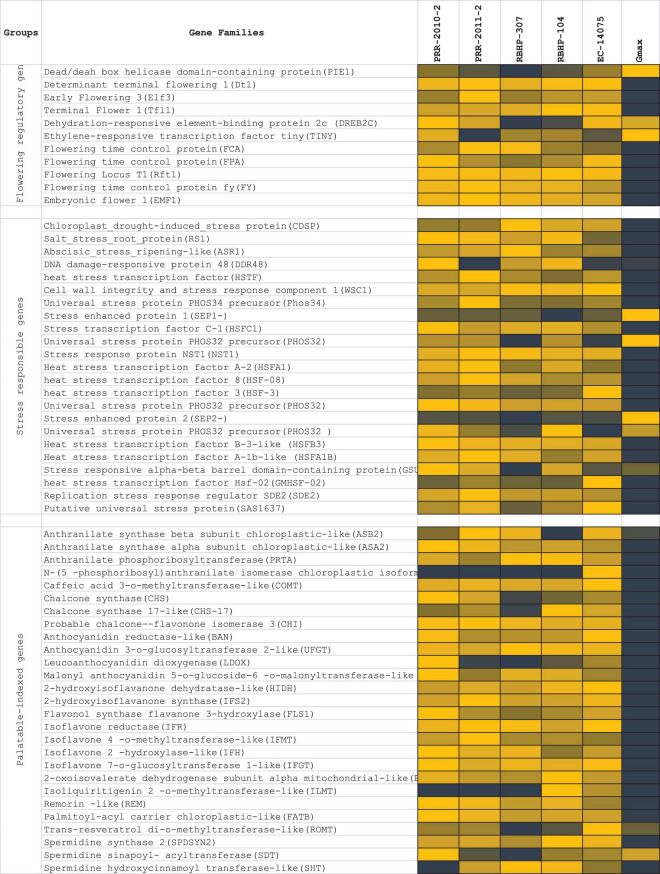
List of genes’ expression selected for late flowering, ligands produced by palatably indexed genes that activates human bitter taste receptors, stress response genes, and disease resistance genes. RNA expressions of five rice bean varieties (log10 of RPKM of total read counts) and de novo assembled RNA expressions of Glycine max was compared for regulations of each functional genes selected for expression profile analysis.

Flowering potential, palatably indexed genes, stress response, and disease resistance genes were the four gene families that were taken into account for screening and expression analysis in the rice bean genome (Figure 9). *Palatability-specific gene pooling:* A total of 27 genes (*ASB2*, *ASA2*, *PRTA*, *PAI2*, *COMT*, *CHS*, *CHS-17*, *CHI*, *BAN*, *UFGT*, *LDOX*, *5MAT*, *HIDH*, *IFS2*, *FLS1*, *IFR*, *IFMT*, *IFH*, *IFGT*, *BCKDHA*, *ILMT*, *REM*, *ATB*, *ROMT*, *SPDSYN2*, *SDT*, and *SHT*) corresponding to closer nodes for bitter metabolite functions (Dagan-Wiener et al., 2019) that are crucial to palatably indexed, which activates taste receptors in human gestations, were retrieved and analyzed in the selected five rice bean varieties (PRR-2010-2, PRR-2011-2, RBHP-307, RBHP-104, and VRB-3) along with Glycine max as reference transcriptome sample (SRA: SRX6788895). We found 3 loci (scaffold-specific) of 27 specific genes expressed in selected reference (*Gmax*). *Flowering-specific gene pooling:* In total, 11 flowering potential genes from selected legume crops were subjected to BLAST search against rice bean draft genome from the assembly and analyzed for transcript expressions; 3 genes were found that are responsible for late flowering, which are expressed high in Glycine max and 8 genes are not expressed in rice bean that encounters the commitment of late flowering of rice bean. *Stress-responsive genes:* A total of 23 stress-responsive gene families found the rice bean predicted genes. A total of 5 gene families out of 23 selected genes [stress enhanced protein 1 (SEP1-), universal stress protein PHOS32 precursor (PHOS32), stress enhanced protein 2 (SEP2-), and stress-responsive alpha-beta barrel domain-containing protein (GSU2970)] were found upregulated in rice bean, wherein it is downregulated in Glycine max. The list of stress sequence coverage, expressions, and the species distribution is provided in Figure 9.

### Validation of flowering and palatability related genes by quantitative real-time polymerase chain reaction

**FIGURE 10:**
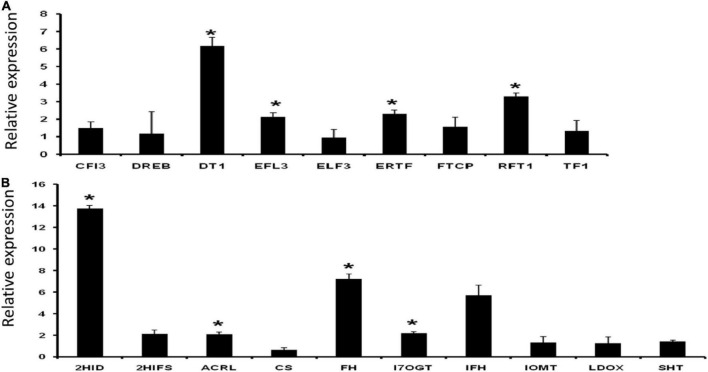
Relative expression of panels **(A)** flowering and **(B)** palatability related genes. Data represent the mean ± SE of 3 replications. Columns marked with an asterisk indicate statistically significant changes, as determined by Student’s *t*-test (*p < 0.05).

A set of nine flowering- and ten palatability-regulating genes (Supplementary Excel 1) were earmarked for validation by qRT-PCR analysis. A total of four flowering-related genes (DT1, EFL3, ERTF, and RFT1) showed significantly higher expressions than normalizer gene 2-oxoisovalerate dehydrogenase expression. Among the ten palatability modulating or related genes for instance, 2HID, ACRL, FH, and I7OGT showed significantly increased expressions (Figure 10).

## Discussion

Rice bean is less familiar among Asiatic *Vigna* varieties but presents higher potential value than adzuki bean, mung bean, and cowpea, which are in the same genetic linkage map in the genome profile. The domestication and subsequent breeding *Vigna umbellata* focusing on its palatability and flowering potential, stress responsive genes, and disease resistance gene families have had inconsistent effects on its genetic diversity. Most varieties of rice bean are highly photoperiod sensitive and are therefore late flowering show strong vegetative growth when grown in the subtropics. The induced mutation of genes based on this study could transform a climbing perennial that develops axillary inflorescences during a juvenile stage of many years into a compact plant with rapid terminal flower and fruit development. Expressions of important flowering-related gene families are found significant in five selected varieties of rice bean by positioning read mapping values with *Glycine max* expression profiles. The early flowering-3 (Elf3) gene found upregulated in rice bean varieties that would increase functional factors of transcriptional coregulation activity (corepressor complex), because of long-term interaction with phytochrome B and nematode feeding. Dt1 and Dt2 genes have been implicated in regulating stem growth habit and hence affect plant’s indeterminate growth (Bernard, 1972; Thompson et al., 1997; Liu et al., 2010). Early flowering 3 (EFL3) regulates circadian clock and photoperiodic flowering (Hicks et al., 2001); whereas RFT1 (FLOWERING LOCUS T1) induces the initiation of the reproductive phase and promotes the expression of major genes that regulate inflorescence development. Data reveal that aforesaid genes Dt1, EFL3, RFT1, and ERTF display significant modulation in expression, thereby exhibiting a strong correlation to its architecture and physiology (Figure 10A). Rice beans are rich source of phenolic acids and flavonoids. In line with our data support the significant enhancement in expression of isoflavone (health boosting compound) pathway genes, such as 2HID (2-hydroxyisoflavanone dehydratase-like), ACRL (anthocyanidin reductase-like), FH (flavanone 3-hydroxylase), and I7OGT (isoflavone 7-O-glucosyltransferase 1-like) (Figure 10B). PIE (photoperiod-independent early flowering) gene families are mediating to regulate FLC-related transcription factors by chromatic remodeling, are downregulated in control group, and are upregulated in rice bean. TFL gene families reported from rice bean control inflorescence identify and maintain indeterminate inflorescences could be a better target for breeding purpose. These genes prevent the expression of APETALA1 and LEAFY, which play major roles in regulating the time of flowering. Regulations of FCA – flowering time control, photoperiod-independent early flowering isoforms to reactivate FLC in early embryos and chromatin modeling based on the transcriptional regulation are the significant source for late flowering targets. Palatability- and digestibility-specific gene pooling increase the understanding of trait qualities that are preferred, including hard seededness, for all genotypes used by farmers in India. Taste modalities are aversive to each species and respondents. A total of 37 genes are screened for palatability indexed ligands that are produced from 8 enzymatic responses. Bitterness is elicited by diverge metabolite products from an enzymatic reaction (Garg et al., 2018). Expression of these genes that are indexed for bitterness is mapped with *Glycine max* as control reference. We found down-regulation of the 15 genes in *G. max* as compared to rice bean which provide the scientific evident that these are the candidate genes that can be targeted to enhance the palatability index of the rice bean domestic cultivars. Ligands from selected/screened enzymatic sources are reported as bitter predictors to limit the threshold of receptor promiscuity. Nissim et al. (2017) reported 270 molecules as bitterness predictors, in which rice bean has 15 bitterness producible ligands that activate taste receptors – T2R in humans. These ligands activating 8 enzymatic reaction that is projected as chemosensory studied proteins for taste receptivity. Expressions of these 15 genes are the indicative markers in Glycine max for bitterness. Anthranilate, chalcone, cyanidin, flavone, morin, palmitoyl, resveratrol, and spermidine are the major functional molecules to determine palatability index of rice bean. These ligands are aversive to act as stimulus and opposed to olfactory. It produced from deamination of coregulated aromatic acids derived by subsequent hydrolytic and reduction by non-enzymatic compositions and storage conditions. Anthranilate N-methyltransferase-like, -synthase alpha subunit, -beta subunit, and -phosphoribosyltransferase are found to have 2-fold expression in *Glycine max* that acts as the taste modifiers (specific to hypersensitivity-induced reaction). Anthranilate basically involves in the synthesis of acridine alkaloids that activates most of T2R (17988223). All other 28 genes, reported for palatability index, are downregulated in *Gmax* and upregulated in rice bean. Pharmacophore of these 14 ligands is mapped to the closer proximity of flavonoid group. These promises, the evolution of these ligands from mapped genes, are self-evident that C-ring of flavonoids is the precursive and crucial for taste receptor activation.

Rice bean genes prevail resistance to biotic and abiotic stresses, which is an issue that is generally addressed in most plant species in the process of domestication and crop improvement. *Vigna umbellata*, we identified 97% of the coding domains of selected gene families, such as those encoding the Rho-type GTPase-activating proteins, serine–threonine protein kinases (TAO1), presynaptic morphology proteins, and enhanced disease resistance 1 isoforms, which limit the initiation of cell death and establish a hypersensitive response.

A total of fourteen medicinally important genomes were compared with the genes of the rice bean assembly. Genes that are abundant provide in-depth transcript profiles of individual genes. This alignment facilitated the identification of candidate genes pertinent to the pathways of interest, as well as non-pathway targets whose expression is consistent with the synthesis of medicinally valuable compounds in rice bean. *Rauwolfia serpentine* contains an ajmalan alkaloid-rich metabolite showing a high gene alignment frequency with rice bean. This metabolite is an anti-arrhythmic in the human system, and a mixture of rapidly interconverting epimers was found.

## Conclusion

The draft of the *Vigna umbellata* genome provides insightful genetic information from our draft genome assembly to domesticate. The explored data from whole genome-sequence and its expression profile document that rice bean is suitable for cultivation in large areas and potentially holds high commercial value. This identified palatable, flowering stress-responsive and medically active gene library offers an avenue for scientists to improve the traits *via* gene editing and farmers to cultivate this legume crop variety harboring immense nutritional potential for extensive consumption, to meet the centuries-long challenge of its cultivation and consumption. This study will bring the nutritionally crucial legume crop into mainstream commercial platform to serve several purposes, including trait improvement for consumption, thereby alleviating the issue of micronutrient malnutrition, prevalent worldwide.

## Accession list of selected species under leguminous crops genome comparison

*Arachis hypogea*: PIVG00000000.1; PRJNA476953, *Cajanus cajan*: AGCT00000000.1; PRJNA376605, *Cicer arietinum:* ANPC00000000.1; PRJNA190909, *Glycine max:* ACUP00000000.3; PRJNA19861,*Lupinus angustifolius*: CM007380.1; PRJNA356456, Mucuna pruriens: QJKJ00000000.1; PRJNA414658, *Phaseolus coccineus*: QBDZ00000000.1; PRJNA448610, *Phaseolus vulgaris:* ANNZ00000000.1; PRJNA240798, *Pisum sativum:* PUCA000000000.1; PRJNA432052, *Vicia faba*: CSVX00000000.1; PRJEB8906, *Vigna angularis:* JZJH00000000.1; PRJNA328963, *Vigna radiata:* JJMO00000000.1; PRJNA301363, and *Vigna unguiculata*: NBOW00000000.1; PRJNA521068.

## Data availability statement

The datasets presented in this study can be found in online repositories. The names of the repository/repositories and accession number(s) can be found in the article/Supplementary material.

## Author contributions

TK proposed the ground idea of working on orphan crops *Vigna umbellata*. ME organized the data to assemble, analyzed and plotted the data, and wrote the manuscript. AT and NR helped to compile the data in the excel for plotting. MN, AM, and BB supported to improve the assembled data in their respective institute server facility. Gayacharan helped to supply the seed. CB helped to sequence and assemble the raw data. RV, SS, KA, and JB helped to collect the literature, library preparation, and language corrections. All authors contributed to the article and approved the submitted version.

## Supplementary material

Supplementary Excel 1 Primer list and qPCR details.

Supplementary Excel 2 SSR analysis report.

Supplementary Excel 3 Enzymes tables.

Supplementary Excel 4 BLASTx analysis complete list.

Supplementary Excel 5 Pathways analysis report.

## References

[B1] Andersen P. Kumar N. Acharya B. K. (2009). *Rice bean food preparation and diets.* Available Online at: https://bora.uib.no/bora-xmlui/handle/1956/6611

[B2] Andersen P. (2012). Challenges for under-utilized crops illustrated by rice bean (Vigna umbellata) inIndia and Nepal. *Int. J. Agric. Sustain*. 10, 164–174.

[B3] Bandillo N. Raghavan C. Muyco P. A. Sevilla M. A. L. Lobina I. T. Dilla- Ermita C. J. (2013). Multi-parent advanced generation inter- cross (MAGIC) populations in rice: Progress and potential for genetics research and breeding. *Rice* 6:11. 10.1186/1939-8433-6-11 PMC4883706 24280183

[B4] Basavaprabhu N. M. Niranjanmurthy N. M. Asif M. Venkatesha K. T. Vijay Kumar K. V. (2013). Genetic divergence analysis in rice bean, *Vigna umbellata* (L.). *Int. J. Plant Sci*. 8, 65–167.

[B5] Basu P. Scholten B. A. (2012). Technological and social dimensions of the Green revolution: Connecting pasts and futures. *Int. J. Agric. Sustain.* 2 109–116. 10.1080/14735903.2012.674674

[B6] Bernard R. L. (1972). Two genes affecting stem termination in soybeans. *Crop Sci.* 12 235–239.

[B7] Borrill P. Harrington S. A. Simmonds J. Uauy C. (2019). Identification of transcription factors regulating senescence in wheat through gene regulatory network modelling. *Plant Physiol.* 3 1740–1755. 10.1104/pp.19.00380 31064813 PMC6752934

[B8] Campbell M. Oakeson K. F. Yandell M. Halpert J. R. Dearing D. (2016). The draft genome sequence and annotation of the desert woodrat *Neotoma lepida*. *Genom. Data* 23 58–59. 10.1016/j.gdata.2016.06.008 27408812 PMC4927542

[B9] Campbell M. S. Holt C. Moore B. Yandell M. (2014). Genome annotation and curation using MAKER and MAKER-P. *Curr. Protoc. Bioinformatics* 48 4.11.1–4.11.39. 10.1002/0471250953.bi0411s48 25501943 PMC4286374

[B10] Cao L. Lu X. Zhang P. Wang G. Wei L. Wang T. (2019). Systematic analysis of differentially expressed maize ZmbZIP genes between drought and rewatering transcriptome reveals bZIP family members involved in abiotic stress responses. *Int. J. Mol. Sci.* 20:4103. 10.3390/ijms20174103 31443483 PMC6747360

[B11] Carette J. E. Verver J. Martens J. van Kampen T. Wellink J. van Kammen A. (2002). Characterization of plant proteins that interact with cowpea mosaic virus ‘6OK’ protein in the yeast two-hybrid system. *J. Gen. Virol.* 83(Pt 4), 885–893. 10.1099/0022-1317-83-4-885 11907339

[B12] Carmena M. Wheelock M. Funabiki H. Earnshaw W. C. (2012). The chromosomal passenger complex (CPC): From easy rider to the godfather of mitosis. *Nat. Rev. Mol. Cell Biol.* 13 789–803. 10.1038/nrm3474 23175282 PMC3729939

[B13] Chen J. Yang L. Yan X. Liu Y. Wang R. Fan T. (2016). Zinc-Finger transcription factor ZAT6 positively regulates cadmium tolerance through the glutathione-dependent pathway in Arabidopsis. *Plant Physiol.* 171 707–719. 10.1104/pp.15.01882 26983992 PMC4854688

[B14] Cheon K. Kim K. Kwak M. Lee B. Yoo K. (2019). The complete chloroplast genome sequences of four *Viola* species (Violaceae) and comparative analyses with its congeneric species. *PLoS One* 14:e0214162. 10.1371/journal.pone.0214162 30893374 PMC6426196

[B15] Clement S. L. Cristofaro M. Cowgill S. E. Weigand S. (2019). “Germplasm resources, insect resistance, and grain legume improvement,” in *Global plant genetic resources for insect-resistant crops*, eds Clement S. L. Quisenberry S. S. (Boca Raton, FL: CRC Press), 131–148. 10.1201/9780429117855

[B16] Comelli P. König J. Werr W. (1999). Alternative splicing of two leading exons partitions promoter activity between the coding regions of the maize homeobox gene Zmhox1a and Trap (transposon-associated protein). *Plant Mol. Biol.* 5 615–625. 10.1023/A:1006382725952 10645721

[B17] Dagan-Wiener A. Di Pizio A. Nissim I. Bahia M. S. Dubovski N. Margulis E. (2019). BitterDB: Taste ligands and receptors database in 2019. *Nucleic Acids Res.* 47 D1179–D1185. 10.1093/nar/gky974 30357384 PMC6323989

[B18] Dahipahle A. V. Kumar S. Sharma N. Singh H. Kashyap S. Meena H. (2019). Rice bean - A multipurpose, underutilized, potential nutritive fodder legume - A review. *J. Pure Appl. Microbiol.* 11 433–439. 10.22207/JPAM.11.1.57

[B19] Dai X. Sinharoy S. Udvardi M. D. Zhao P. X. (2013). Plant TFcat: An online plant transcription factor and transcriptional regulator categorization and analysis tool. *BMC Bioinformatics* 14:321. 10.1186/1471-2105-14-321 24219505 PMC4225725

[B20] Darling A. E. Mau B. Perna N. T. (2010). progressiveMauve: Multiple genome alignment with gene gain, loss and rearrangement. *PLoS One* 5:e11147. 10.1371/journal.pone.0011147 20593022 PMC2892488

[B21] De Vega J. J. Ayling S. Hegarty M. Kudrna D. Goicoechea J. L. Ergon Å. (2015). Red clover (*Trifolium pratense* L.) draft genome provides a platform for trait improvement. *Sci. Rep.* 5:17394. 10.1038/srep17394 26617401 PMC4663792

[B22] De Vetten N. C. Ferl R. J. (1995). Characterization of a maize G-box binding factor that is induced by hypoxia. *Plant J.* 7 589–601. 10.1046/j.1365-313x.1995.7040589.x 7742856

[B23] Dhiman J. S. Kang M. S. Parshad V. R. Khanna P. K. Bal S. S. Gosal S. S. (2010). Improved seeds and green revolution. *J. New Seeds* 11 65–103. 10.1080/1522886X.2010.481777

[B24] Dong X. Stothard P. Forsythe I. J. Wishart D. S. (2004). PlasMapper: A web server for drawing and auto-annotating plasmid maps. *Nucleic Acids Res.* 32 W660–W664. 10.1093/nar/gkh410 15215471 PMC441548

[B25] FAOSTAT (2019). *Food and agriculture organization of the United Nations. Food and agriculture data*. Available online at: https://www.fao.org/faostat/

[B26] Friedrich J. K. Panov K. I. Cabart P. Russell J. Zomerdijk J. C. (2005). TBP-TAF complex SL1 directs RNA polymerase I pre-initiation complex formation and stabilizes upstream binding factor at the rDNA promoter. *J. Biol. Chem.* 280 29551–29558. 10.1074/jbc.M501595200 15970593 PMC3858828

[B27] Garg N. Sethupathy A. Tuwani R. Nk R. Dokania S. Iyer A. (2018). FlavorDB: A database of flavor molecules. *Nucleic Acids Res.* 46 D1210–D1216. 10.1093/nar/gkx957 29059383 PMC5753196

[B28] Gaur P. M. Varshney R. K. Upadhyaya H. D. Vadez V. Sharma K. K. Bhatnagar P. (2018). Advances in food legumes research at ICRISAT. *Ethiopian J. Crop Sci.* 6 1–47.

[B29] George Thompson A. M. Lancu C. V. Nguyen T. T. H. Kim D. Choe J. (2015). Inhibition of human GLUT1 and GLUT5 by plant carbohydrate products; insights into transport specificity. *Sci. Rep.* 5:12804. 10.1038/srep12804 26306809 PMC4549712

[B30] Gibbs R. A. Weinstock G. M. Metzker M. L. Muzny D. M. Sodergren E. J. Scherer S. (2004). Rat genome sequencing project consortium. genome sequence of the brown Norway rat yields insights into mammalian evolution. *Nature* 428 493–521. 10.1038/nature02426 15057822

[B31] Graham P. H. Vance C. P. (2003). Legumes: Importance and constraints to greater use. *Plant Physiol.* 3 872–877. 10.1104/pp.017004 12644639 PMC1540286

[B32] Hicks K. A. Albertson T. M. Wagner D. R. (2001). EARLY FLOWERING3 encodes a novel protein that regulates circadian clock function and flowering in Arabidopsis. *Plant Cell* 13 1281–1292. 10.1105/tpc.13.6.1281 11402160 PMC135582

[B33] Ho C. L. Lee W. Lim E. L. (2018). Unraveling the nuclear and chloroplast genomes of an agar producing red macroalga, *Gracilaria changii* (Rhodophyta, Gracilariales). *Genomics* 110 124–133. 10.1016/j.ygeno.2017.09.003 28890206

[B34] Hwang J. J. Chambon P. Davidson I. (1993). Characterization of the transcription activation function and the DNA binding domain of transcriptional enhancer factor-1. *EMBO J.* 12 2337–2348. 10.1002/j.1460-2075.1993.tb05888.x 8389695 PMC413464

[B35] Jain M. Misra G. Patel R. K. Priya P. Jhanwar S. Khan A. W. (2013). A draft genome sequence of the pulse crop chickpea (*Cicer arietinum* L.). *Plant J.* 74 715–729. 10.1111/tpj.12173 23489434

[B36] Joshi K. D. Bhandari B. Gautam R. Bajracharya J. Hollington P. (2007). “Ricebean, a multipurpose underutilized legume,” in *Paper presented at the 5th international symposium on new crops and uses, their role in a rapidly changing world*, (Southampton: The University of Southampton).

[B37] Joshi R. Wani S. H. Singh B. Bohra A. Dar Z. A. Lone A. A. (2016). Transcription factors and plants response to drought stress: Current understanding and future directions. *Front. Plant Sci.* 7:1029. 10.3389/fpls.2016.01029 27471513 PMC4943945

[B38] Kanehisa M. Sato Y. (2020). KEGG Mapper for inferring cellular functions from protein sequences. *Protein Sci.* 29 28–35. 10.1002/pro.3711 31423653 PMC6933857

[B39] Kang Y. Satyawan D. Shim S. Lee T. Lee J. Hwang W. (2015). Draft genome sequence of adzuki bean, *Vigna angularis*. *Sci. Rep.* 5:8069. 10.1038/srep08069 25626881 PMC5389050

[B40] Kang Y. J. Kim S. K. Kim M. Y. Lestari P. Kim K. H. Ha B. K. (2014). Genome sequence of mungbean and insights into evolution within Vigna species. *Nat. Commun.* 11:5443. 10.1038/ncomms6443 25384727 PMC4241982

[B41] Keller O. Kollmar M. Stanke M. Waack S. (2011). A novel hybrid gene prediction method Employing protein multiple sequence alignments. *Bioinformatics* 27 757–763. 10.1093/bioinformatics/btr010 21216780

[B42] Kim J. Daadi M. M. (2019). Bioinformatics analysis of single-cell RNA-Seq raw data from iPSC-derived neural stem cells. *Methods Mol. Biol*. 1919 145–159. 10.1007/978-1-4939-9007-8_11 30656627 PMC6605033

[B43] Korf I. (2004). Gene finding in novel genomes. *BMC Bioinformatics* 5:59. 10.1186/1471-2105-5-59 15144565 PMC421630

[B44] Krishna M. S. R. Sokka Reddy S. Satyanarayana S. D. V. (2017). Marker-assisted breeding for introgression of opaque-2 allele into elite maize inbred line BML-7. *3 Biotech* 7:165. 10.1007/s13205-017-0842-2 28660457 PMC5489451

[B45] Kumar A. Sheoran N. Prakash G. Ghosh A. Chikara S. K. Rajashekara H. (2017). Genome sequence of a unique *Magnaporthe oryzae* RMg-Dl isolate from India that causes blast disease in diverse cereal crops, obtained using PacBio single-molecule and Illumina HiSeq2500 sequencing. *Genome Announc.* 5:e01570-16. 10.1128/genomeA.01570-16 28209817 PMC5313609

[B46] Latef A. Ahmad P. (2015). “Legumes and breeding under abiotic stress: An overview,” in *Legumes under environmental stress*, eds Azooz M. M. Ahmad P. (Hoboken, NJ: John Wiley & Sons Ltd), 1–20.

[B47] Liu B. H. Watanabe S. Uchiyama T. Kong F. J. Kanazawa A. Xia Z. J. (2010). The soybean stem growth habit gene Dt1 is an ortholog of Arabidopsis *TERMINAL FLOWER1*. *Plant Physiol.* 153 198–210. 10.1104/pp.109.150607 20219831 PMC2862436

[B48] Medicinal Plant Genomics Resource [MPGR] (2019). *Medicinal Plant Genomics Resource. Medicinalplantgenomics.msu.edu.* Available Online at: http://mpgr.uga.edu/

[B49] Mochida K. Sakurai T. Seki H. Yoshida T. Takahagi K. Sawai S. (2017). Draft genome assembly and annotation of *Glycyrrhiza uralensis*, a medicinal legume. *Plant J.* 89 181–194. 10.1111/tpj.13385 27775193

[B50] Morgat A. Lombardot T. Coudert E. Axelsen K. Neto T. B. Gehant S. (2020). Enzyme annotation in UniProtKB using Rhea. *Bioinformatics* 36 1896–1901. 10.1093/bioinformatics/btz817 31688925 PMC7162351

[B51] Muñoz-Amatriaín M. Mirebrahim H. Xu P. Wanamaker S. I. Luo M. Alhakami H. (2017). Genome resources for climate-resilient cowpea, an essential crop for food security. *Plant J.* 89 1042–1054. 10.1111/tpj.13404 27775877

[B52] Nakamura T. Yamada K. D. Tomii K. Katoh K. (2018). Parallelization of MAFFT for large-scale multiple sequence alignments. *Bioinformatics* 34 2490–2492. 10.1093/bioinformatics/bty121 29506019 PMC6041967

[B53] Nissim I. Dagan-Wiener A. Niv M. Y. (2017). The taste of toxicity: A quantitative analysis of bitter and toxic molecules. *IUBMB Life* 69, 938–946. 10.1002/iub.1694 29130618

[B54] Ng N. (2013). “Conserving tropical leguminous food crops,” in *Conservation of tropical plant species*, eds Normah M. Chin H. Reed B. (New York, NY: Springer), 213–247.

[B55] OMGenomics (2019). *OMGenomics – Bioinformatics software.* Available Online at: https://omgenomics.com/circa/

[B56] Pattanayak A. Roy S. Sood S. Iangrai B. Banerjee A. Gupta S. (2019). Rice bean: A lesser known pulse with well-recognized potential. *Planta* 250 873–890. 31134340 10.1007/s00425-019-03196-1

[B57] Peng X. Liu H. Chen P. Tang F. Hu Y. Wang F. (2019). Chromosome-Scale genome assembly of paper mulberry (*Broussonetia papyrifera*) provides new insights into its forage and papermaking usage. *Mol. Plant* 12 661–677. 10.1016/j.molp.2019.01.021 30822525

[B58] Rana J. Sood S. Gupta A. Negi K. S. Lal H. (2014). Rice bean variety VRB-3 (Him Shakti). *Indian J. Genet. Plant Breed.* 74:268.

[B59] Rügner A. Frohnmeyer H. Näke C. Wellmer F. Kircher S. Schäfer E. (2001). Isolation and characterization of four novel parsley proteins that interact with the transcriptional regulators CPRF1 and CPRF2. *Mol. Genet. Genomics* 265 964–976. 10.1007/s004380100502 11523788

[B60] Shi P. Wu T. Li P. Guo B. Fang G. Dong Y. (2017). Use of processed data to design an orderly logic gate to construct plasmids in GenoCAD. *IET Syst. Biol.* 2 65–68. 10.1049/iet-syb.2016.0043 28476974 PMC8687292

[B61] Singh C. R. He H. Ii M. Yamamoto Y. Asano K. (2004). Efficient incorporation of eukaryotic initiation factor 1 into the multifactor complex is critical for formation of functional ribosomal preinitiation complexes in vivo. *J. Biol. Chem.* 279 31910–31920. 10.1074/jbc.M313940200 15145951

[B62] Singh N. K. Gupta D. K. Jayaswal P. K. Mahato A. K. Dutta S. Singh S. (2012). The first draft of the pigeonpea genome sequence. *J. Plant Biochem. Biotechnol.* 1 98–112. 10.1007/s13562-011-0088-8 24431589 PMC3886394

[B63] Smathers C. M. Robart A. R. (2019). The mechanism of splicing as told by group II introns, Ancestors of the spliceosome. *Biochim. Biophys. Acta Gene Regul. Mech.* 1862:194390. 10.1016/j.bbagrm.2019.06.001 31202783

[B64] Takahashi Y. Iseki K. Kitazawa K. Muto C. Somta P. Irie K. (2015). A homoploid hybrid between wild *Vigna* species found in a limestone karst. *Front. Plant Sci.* 6:1050. 10.3389/fpls.2015.01050 26648953 PMC4664699

[B65] Takase H. Minami M. Iwabuchi M. (1991). Sequence-specific single-strand DNA-binding proteins that interact with the regulatory regions of wheat histone H3 and H4 genes. *Biochem. Biophys. Res. Commun.* 176 1593–1600. 10.1016/0006-291x(91)90470-r 2039533

[B66] Tarailo-Graovac M. Chen N. (2009). Using repeat masker to identify repetitive elements in genomic sequences. *Curr. Protoc. Bioinformatics* 4 10.1002/0471250953.bi0410s25 19274634

[B67] Thompson J. A. Bernard R. L. Nelson R. L. (1997). A third allele at soybean dt1 locus. *Crop Sci.* 37 757–762. 10.2135/cropsci1997.0011183X003700030011x

[B68] Tomooka N. (2009). “The origin of rice bean (*Vigna umbellata*) and azuki bean (*V. angularis*): The evolution of two lesser-known Asian beans,” in *An illustrated eco-history of the Mekong River Basin*, ed. Akimichi T. (Bangkok: White Lotus Co).

[B69] Urbancsok J. Bones A. Kissen R. (2018). Benzyl cyanide leads to auxin-like effects through the action of nitrilases in *Arabidopsis thaliana*. *Front. Plant Sci.* 9:1240. 10.3389/fpls.2018.01240 30197652 PMC6117430

[B70] VanBuren R. Bryant D. Edger P. Tang H. Burgess D. Challabathula D. (2015). Single-molecule sequencing of the desiccation-tolerant grass *Oropetium thomaeum*. *Nature* 527 508–511. 10.1038/nature15714 26560029

[B71] Varshney R. Chen W. Li Y. Bharti A. K. Saxena R. K. Schlueter J. A. (2011). Draft genome sequence of pigeonpea (*Cajanus cajan*), an orphan legume crop of resource-poor farmers. *Nat. Biotechnol.* 30 83–89. 10.1038/nbt.2022 22057054

[B72] Vining K. J. Johnson S. R. Ahkami A. Lange I. Parrish A. N. Trapp S. C. (2017). Draft genome sequence of *Mentha longifolia* and development of resources for mint cultivar improvement. *Mol. Plant* 10 323–339. 10.1016/j.molp.2016.10.018 27867107

[B73] Wang M. Wang Z. Mao Y. Lu Y. Yang R. Tao X. (2019). Optimizing base editors for improved efficiency and expanded editing scope in rice. *Plant Biotechnol. J.* 17 1697–1699. 10.1111/pbi.13124 30963683 PMC6686124

[B74] Waterhouse R. M. Seppey M. Simao F. A. Manni M. Ioannidis P. Klioutchnikov G. (2017). BUSCO applications from quality assessments to gene prediction and phylogenomics. *Mol. Biol. Evol.* 35 543–548. 10.1093/molbev/msx319 29220515 PMC5850278

[B75] Xu C. Jiao C. Sun H. Cai X. Wang X. Ge C. (2017). Draft genome of spinach and transcriptome diversity of 120 Spinacia accessions. *Nat. Commun*. 8:15275. 10.1038/ncomms15275 28537264 PMC5458060

[B76] Zhang Y. Massel K. Godwin I. D. (2018). Applications and potential of genome editing in crop improvement. *Genome Biol.* 19:210. 10.1186/s13059-018-1586-y 30501614 PMC6267055

[B77] Zhao Q. Yang J. Cui M. Y. Liu J. Fang Y. Yan M. (2019). The reference genome sequence of *Scutellaria baicalensis* provides insights into the evolution of wogonin biosynthesis. *Mol. Plant* 12 935–950. 10.1016/j.molp.2019.04.002 30999079

[B78] Zhao Y. Sha W. Wang Q. Y. Zhai Y. Zhao Y. Shao S. L. (2015). Molecular cloning and activity analysis of a seed-specific FAD2-1B gene promoter from *Glycine max*. *Cell. Mol. Biol.* 61 85–89. 10.14715/cmb/2015.61.4.14 26386665

[B79] Zhuang W. Chen H. Yang M. Wang J. Pandey M. K. Zhang C. (2019). The genome of cultivated peanut provides insight into legume karyotypes, polyploid evolution and crop domestication. *Nat. Genet.* 51 865–876. 10.1038/s41588-019-0402-2 31043757 PMC7188672

