# Supplementary material for: De novo genome assembly of rice bean (Vigna umbellata) – A nominated nutritionally rich future crop reveals novel insights into flowering potential, habit, and palatability centric – traits for efficient domestication
Source: Front Plant Sci. 2022 Oct 4;13:739654. doi: 10.3389/fpls.2022.739654 (PMC9577371; doi:10.3389/fpls.2022.739654)
Supplement: Supplementary file 2 [file Data_Sheet_2.docx]

**De novo genome assembly of Rice Bean (*Vigna umbellata*) - a nominated nutritionally rich future crop reveals novel insights into flowering potential, habit and palatability centric - traits for efficient domestication**

Tanushri Kaul^*1^, Murugesh Easwaran^#1^, Arulprakash Thangaraj^1^, Arun Meyyazhagan^2^, Mamta Nehra^1^, Nitya Meenakshi Raman^1^, Rachana Verma^1^, Sonia Khan Sony^1^, Khaled Fathy Abdel^1^, Jyotsna Bharti^1^, Gayacharan I ^3^, Chandan Badapanda^4^, Balamuralikrishnan Balasubramanian^5^.

1. Nutritional Improvement of Crops, International Centre for Genetic Engineering and Biotechnology, New Delhi. India - 110070
2. Euroespes Biomedical Research Center, Institute of Medical Science and Genomic Medicine, Bergondo – 15165, La Corunna, Spain
3. ICAR- National Bureau of Plant Genetic Resources, PUSA Campus, New Delhi – 110012
4. Xcelris Labs Limited, Ahmedabad – 380015
5. Department of Food Science and Biotechnology, College of Life Sciences, Sejong University, Seoul-05006, South Korea.

#Equal contribution

*Corresponding author : tanushri@icgeb.res.in

Prof. Tanushri Kaul

Group Leader

Nutritional Improvement in Crops Group

Plant Biology and Biotechnology Division

International Centre for Genetic Engineering and Biotechnology

New Delhi, India

Phone: +91-11-26742962 (D); +91-11-26741007 (Ext 373); Mobile: 9999966205

http://www.icgeb.org/nutritional-improvement-of-crops.html

http://www.icgeb.org/tanushri-kaul.html

**SUPPLEMENTARY TABLES**

| **Statistics composition** | **Allpath-LG (primary)** | **Allpath-LG-Gapclosure** | **PBjelly** | **Pbjelly-gapclosure** |
| --- | --- | --- | --- | --- |
| No. of scaffolds | 17601 | 17601 | 15521 | 15521 |
| No. of contigs | 41447 | 39387 | 36461 | 36241 |
| Total genome length including gaps | 389810950 (bp) | 387954823 (bp) | 414706990 (bp) | 414646213 (bp) |
| Total genome length without gaps | 301496373 (bp) | 351493463 (bp) | 382473710 (bp) | 387569263 (bp) |
| Average scaffold size including gaps | 22147 | 22042 | 26719 | 26715 |
| Scaffold N50 | 65704 | 65252 | 78103 | 78112 |
| Maximum scaffold size | 563916 (bp) | 564527 (bp) | 1043617 (bp) | 1042625 (bp) |
| Minimum scaffold size | 882 (bp) | 882 (bp) | 886 (bp) | 886 (bp) |

Table 1: Allpath-LG gapclosure and Pbjelly gapclosure comparison of Summation and assembly statistical composition from rice bean genome assembly.

|  | **Scaffold Size** | **Scaffold No** | **Size of Assembly** |
| --- | --- | --- | --- |
| **N50** | 78112 | 7652 | 207323106 |
| N75 | 33596 | 12330 | 310981659 |
| N90 | 14991 | 12767 | 373189888 |

Table 2: Scaffold summation (N50, N75 and N90) distribution of rice bean assembled data.

| **Length distribution of Assembly** | **Count** |
| --- | --- |
| >300 & <=900 | 10 |
| >900&<=1000 | 256 |
| >1000&<=2000 | 4095 |
| >2000&<=4000 | 1519 |
| >4000&<=6000 | 803 |
| >6000&<=8000 | 625 |
| >8000 & <=10000 | 779 |
| >10000 | 7434 |

Table 3: Length distribution of genome assembly by Allpath-LG with discrete distribution intervals.

| **Length Distribution of Genes Predicted** | **Total bp** |
| --- | --- |
| Maximum | 15628 |
| >10000 | 14 |
| >8000 & <=10000 | 22 |
| >6000&<=8000 | 156 |
| >4000&<=6000 | 794 |
| >2000&<=4000 | 7372 |
| >1000&<=2000 | 10362 |
| >300&<=1000 | 10396 |

Table 4: Length distribution of mapped genes with discrete distribution intervals.

| **Statistics composition** | ***Vigna umbellata*** | ***Vigna radiata*** | ***Vigna angularis*** |
| --- | --- | --- | --- |
| Total sequencing data | 69 Gb | Not mentioned | 291 Gb |
| No. of scaffolds | 15521 | 2748 | 3883 |
| No. of contigs | 36241 | 25922 | 36516 |
| Total genome length including gaps | 414646213 (bp) | 463143055 (bp) | 443436934 (bp) |
| Average scaffold size including gaps | 26715 | Not mentioned | Not mentioned |
| Scaffold N50 | 78112 | 1516 | 703 |
| Maximum scaffold size(in bps) | 1042625 | Not mentioned | Not mentioned |
| Minimum scaffold size(in bps) | 886 | 1000 | 1000 |

Table 5: Summation comparison of rice bean, mung bean and adzuki bean concerning total sequenced data and N50 scaffold distribution

| **Read Profile** | **Length** |
| --- | --- |
| Assembly reads | 414646213 (bp) |
| Genes predicted | 49811559 (bp) |
| Genes Predicted vs *Vang* bp | 105415055 (bp) |
| Genes Predicted vs *Vrad* bp | 92715800 (bp) |
| Genes Predicted vs *Vung* | 81760762 (bp) |

Table 6: Base pair distribution of Assembled reads: 31276 genes predicted from the assembly, bp of sequence mapped between genes predicted from rice bean and complete genome of adzuki bean, between genes predicted from rice bean and mung bean and between genes predicted from rice bean and cowpea.

| **Read Alignment Profile** | **Counts** |
| --- | --- |
| Total read aligned with Ref *Vang* | 41525 |
| Total read aligned with Ref *Vrad* | 32657 |
| Total read aligned with Ref *Vung* | 16614 |
| Total read aligned for gene annotation with Ref *Vang* | 21006 |
| Total read aligned for gene annotation with Ref *Vrad* | 21657 |
| Total read aligned for gene annotation with Ref *Vung* | 20664 |

Table 7: Total read count distribution of adzuki bean, mung bean and cowpea mapped with assembled reads. Total read count distribution of genes mapped from assembly with adzuki bean, mung bean and cowpea

| **Gene Profile** | **Counts** |
| --- | --- |
| Total Genes Predicted | 31276 |
| Orthologous genes - Genes Predicted Vs Vang | 26001 |
| Orthologous genes - ( Genes Predicted vs Vang ) Vs Ref_Vang | 16892 |
| Orthologous genes - ( Genes Predicted vs Vrad ) Vs Ref_Vrad | 19640 |
| Orthologous genes - ( Genes Predicted vs Vung ) Vs Ref_Vung | 17989 |
| CDS match Genes Predicted with Ref_Vang | 8308 |
| CDS match Genes Predicted with Ref_Vrad | 8985 |
| CDS match Genes Predicted with Ref_Vung | 8515 |

Table 8: Total read counts of total genes predicted from the assembly, read count of orthologous genes compared between mapped genes from assembly and complete reference genome of *Vigna angularis*; Read count of orthologous genes aligned between genes mapped with adzuki bean versus complete reference genome of adzuki bean, between genes mapped with mung bean versus complete reference genome of mung bean, and between genes mapped with cowpea versus complete reference genome of cowpea; CDS read counts between genes predicted and complete reference genome of adzuki bean, mung and cowpea.

| **Aminoacids** | **Codons** | **No. of Codons** |
| --- | --- | --- |
| Ala | TGC | 35 |
| Ala | AGC | 22 |
| Ala | CGC | 15 |
| Arg | TCT | 15 |
| Arg | ACG | 12 |
| Arg | CCG | 9 |
| Arg | CCT | 9 |
| Arg | TCG | 6 |
| Asn | GTT | 38 |
| Asp | GTC | 26 |
| Asp | ATC | 1 |
| Cys | GCA | 16 |
| Gln | CTG | 11 |
| Gln | TTG | 9 |
| Glu | CTC | 19 |
| Glu | TTC | 16 |
| Gly | TCC | 26 |
| Gly | GCC | 23 |
| Gly | CCC | 9 |
| His | GTG | 16 |
| Ile | AAT | 28 |
| Ile | TAT | 10 |
| Leu | AAG | 49 |
| Leu | CAA | 16 |
| Leu | TAG | 10 |
| Leu | TAA | 9 |
| Leu | CAG | 5 |
| Lys | TTT | 26 |
| Lys | CTT | 21 |
| Met | CAT | 50 |
| Phe | GAA | 32 |
| Pro | TGG | 93 |
| Pro | AGG | 50 |
| Pro | CGG | 6 |
| Ser | GCT | 20 |
| Ser | AGA | 19 |
| Ser | TGA | 12 |
| Ser | CGA | 6 |
| Ser | GGA | 2 |
| Thr | AGT | 12 |
| Thr | TGT | 8 |
| Thr | CGT | 5 |
| Trp | CCA | 18 |
| Tyr | GTA | 22 |
| Val | AAC | 23 |
| Val | CAC | 17 |
| Val | TAC | 15 |

Table 9: tRNA prediction response to aminoacid combinations and codon distributions.

| **Scaffolds no** | **tRNA bound region** | **Intronic Spacer** |
| --- | --- | --- |
| Scaffold_12233 | 84 | 20 |
| Scaffold_12233 | 83 | 20 |
| Scaffold_1253 | 93 | 19 |
| Scaffold_12233 | 84 | 19 |
| Scaffold_1253 | 91 | 18 |
| Scaffold_1253 | 91 | 18 |
| Scaffold_1253 | 91 | 18 |
| Scaffold_12841 | 88 | 18 |
| Scaffold_14143 | 84 | 18 |
| Scaffold_14143 | 84 | 16 |
| Scaffold_1708 | 87 | 15 |
| Scaffold_1882 | 83 | 15 |
| Scaffold_2070 | 93 | 14 |
| Scaffold_2144 | 87 | 14 |
| Scaffold_2367 | 91 | 13 |
| Scaffold_2491 | 84 | 12 |
| Scaffold_291 | 84 | 11 |
| Scaffold_4151 | 96 | 10 |
| Scaffold_3448 | 89 | 10 |
| Scaffold_5347 | 87 | 10 |
| Scaffold_4032 | 84 | 10 |
| Scaffold_4151 | 84 | 10 |
| Scaffold_447 | 84 | 10 |
| Scaffold_6052 | 84 | 10 |
| Scaffold_613 | 84 | 10 |
| Scaffold_8061 | 84 | 10 |
| Scaffold_3928 | 83 | 10 |
| Scaffold_5646 | 83 | 10 |
| Scaffold_5299 | 77 | 10 |
| Scaffold_4237 | 76 | 10 |
| Scaffold_8202 | 92 | 9 |
| Scaffold_8995 | 91 | 6 |
| Scaffold_9452 | 86 | 4 |
| Scaffold_995 | 93 | 3 |
| Scaffold_2070 | 104 | **0** |

Table 10: Bound region distributions and intronic spacer distributions of predicted tRNA sequence

| **Seqname** | **Feature start** | **End** | **Score** | **Strand** | **Frame** | **Attribute** | **Bp length** |
| --- | --- | --- | --- | --- | --- | --- | --- |
| Scaffold_8716 | 20911 | 25479 | 1011.3 | - | 0 | 28s_rRNA | 4568 |
| Scaffold_4119 | 4036 | 5842 | 1528.1 | - | 0 | 18s_rRNA | 1806 |
| Scaffold_7914 | 760 | 878 | 30.4 | - | 0 | 8s_rRNA | 118 |
| Scaffold_7914 | 2324 | 2440 | 41.6 | - | 0 | 8s_rRNA | 116 |
| Scaffold_7914 | 6181 | 6297 | 48.4 | - | 0 | 8s_rRNA | 116 |
| Scaffold_7914 | 2110 | 2225 | 56.7 | - | 0 | 8s_rRNA | 115 |
| Scaffold_7914 | 1472 | 1586 | 55.2 | - | 0 | 8s_rRNA | 114 |
| Scaffold_4726 | 53971 | 54085 | 24.8 | + | 0 | 8s_rRNA | 114 |
| Scaffold_7914 | 5249 | 5359 | 19.9 | - | 0 | 8s_rRNA | 110 |
| Scaffold_7914 | 2648 | 2757 | 29.2 | - | 0 | 8s_rRNA | 109 |
| Scaffold_7914 | 3294 | 3400 | 39.8 | - | 0 | 8s_rRNA | 106 |

Table 11: Complete rRNA sequence predicted from scaffolds assembled raw reads. 4 scaffolds ( Scaffold 8716, 4119, 7914 and 4726) are involved in the prediction of 11 rRNA sequence for the distribution of 28s, 18s, 8s subunits.

| **Functions** | **Fragment count** | **Total count** |
| --- | --- | --- |
| EC.1_Oxidoreductases | 64 | 8550 |
| EC.2_Transferases | 59 | 1017 |
| EC.3_Hydrolases | 41 | 683 |
| EC.4_Lyases | 5 | 8 |
| EC.5_Isomerases | 2 | 19 |
| Binding | 194 | 4189 |
| Transporter | 38 | 522 |
| Channel | 13 | 87 |
| Dogmatic | 7 | 88 |
| Inhibition and activation | 40 | 855 |

Table 12: Functions of genes mapped from rice bean distributed for its scaffold count and total number of count. Complete distributions are concerning enzyme classifications and other functional activity of the mapped genes.

| **Gene name expansion** | **TF** | **Vang** | **Vrad** | **Vung** |
| --- | --- | --- | --- | --- |
| 60K protein | 60K protein | 69389 | 60727 | 50914 |
| TEA/ATTS transcription factor | abaA | 2338 | 2366 | 2277 |
| putative chromosomal passenger protein | CPC1 | 1980 | 13824 | 10692 |
| common plant regulatory factor 1-like | CPRF-1 | 15597 | 1830 | 2090 |
| G-box-binding factor | GBF | 159 | 147 | 292 |
| GT1 facilitative glucose transporter GT1 | GT-1 | 1042 | 1010 | 950 |
| GT2 facilitative glucose transporter GT2 | GT-2 | 441 | 369 | 405 |
| hexamer motif 5'-ACGTCA-3 | HBP-1a(c14) | 293575 | 249399 | 204585 |
| Nitrilase Family Member 2 | NIT2 | 49403 | 45274 | 39537 |
| opaque endosperm 2 | Opaque-2 | 25952 | 22118 | 15832 |
| Nuclear factor | SEF4 | 15594 | 14424 | 12870 |
| Sequence-specific single-strand DNA-binding proteins-2 | ssDBP-2 | 352 | 451 | 319 |
| TATA-box binding protein associated factor 1 | TAF-1 | 892 | 930 | 1002 |
| yeast ER-intramembrane protease | YPF1 | 10062 | 9227 | 8848 |
| putative homeodomain-like transcription factor superfamily protein | Zmhox1a | 539 | 524 | 369 |

Table 13: Genes involved in transcription factors in rice bean genes mapped from adzuki bean, mung bean and cowpea.

| **Functions of Genes** | **Vang_Genes** | **Counts** | **Vrad_Genes** | **Counts** | **Vung_Genes** | **Counts** |
| --- | --- | --- | --- | --- | --- | --- |
| SV40_late_19s_int | SV40_late_19s_int | 7049 | SV40_late_19s_int | 6,630 | SV40_late_19s_int | 5,678 |
| Violaxanthin deepoxidase | VDE | 3916 | VDE | 3,527 | VDE | 3,163 |
| T7_gene10_leader | T7_gene10_leader | 3637 | T7_gene10_leader | 3,199 | T7_gene10_leader | 2,808 |
| caspase 3 | casp-3 | 3445 | TEV | 2,919 | TEV | 2,632 |
| hoi-polloi | HOI | 3244 | minicis | 2,781 | SV40_int | 2,405 |
| Tobacco etch virus | TEV | 2971 | SV40_int | 2,640 | minicis | 2,347 |
| ribosomal binding site | RBS_Kozak | 2855 | HIV-1_psi_pack | 2,454 | HIV-1_psi_pack | 2,141 |
| simian virus | SV40_int | 2811 | casp-3 | 2,339 | delta_U3 | 2,067 |
| acid phosphatase Pho1 | PHO1 | 2526 | RBS_Kozak | 2,150 | T7_transl_en_RBS | 1,972 |
| terminator 7 | T7_transl_en_RBS | 2398 | delta_U3 | 2,143 | AraI1I2 | 1,955 |
| Self-inactivating 3' long terminal repeat | delta_U3 | 2276 | T7_transl_en_RBS | 2,110 | casp-3 | 1,948 |
| 2 AraC Arabinose Komplexe | AraI1I2 | 2176 | AraI1I2 | 2,096 | RBS_Kozak | 1,945 |
| inclusion membrane protein A | incA | 2131 | incA | 2,014 | incA | 1,733 |
| protein kinase cAMP-activated catalytic subunit alpha | kemptide_targ | 1967 | kemptide_targ | 1,705 | kemptide_targ | 1,546 |
| flipping DNA recombinase target | FRT | 1861 | FRT | 1,619 | CX_lead | 1,538 |
| ethanolamine kinase | EK | 1349 | myr | 1,218 | myr | 981 |
| myrosinase | myr | 1317 | EK | 1,116 | loxH | 832 |
| protein-lysine 6-oxidase H gene | loxH | 913 | loxH | 878 | EK | 769 |
| protein-lysine 6-oxidase P gene | loxH; loxP | 851 | loxH; loxP | 688 | loxH; loxP | 673 |
| protein-lysine 6-oxidase P gene | loxP | 829 | loxP | 678 | loxP | 653 |
| catabolite activator protein | CAP_BS | 560 | CAP_BS | 518 | CAP_BS | 548 |
| Sex-lethal interactor recombinase | SIN | 499 | SIN | 458 | SIN | 402 |
| thrombin | thromb_targ | 473 | thromb_targ | 370 | loxP; loxH | 363 |
| protein-lysine 6-oxidase P gene | loxP; loxH | 352 | loxP; loxH | 354 | thromb_targ | 338 |
| Acyl carrier protein phosphodiesterase | 3_AcPH | 349 | 3_AcPH | 297 | 3_AcPH | 237 |
| transposons7 | Tn7_att | 325 | Tn7_att | 292 | Tn7_att | 230 |
| 3X Specificty protein 1 | 3xSp1 | 300 | modSV40_late_16s_int | 200 | modSV40_late_16s_int | 193 |
| cohesive end site N | cosN | 130 | cosN | 124 | cosN | 102 |
| luminescence regulator | LITR | 73 | LITR | 62 | LITR | 61 |
| repressor of iron transport | RITR | 73 | RITR | 62 | RITR | 61 |
| Molybdenum import ATP-binding protein | MODC | 2 | LAAV-2_ITR | 2 | LAAV-2_ITR | 2 |
| Encapsulin | Encap | 1 | RAAV-2_ITR | 2 | RAAV-2_ITR | 2 |
| putative transmembrane protein | LAAV-2_ITR | 0 | MODC | 1 | trans_en | 1 |

Table 14: Vector specified 33 gene distribution of rice bean mapped genes from adzuki bean, mung bean and cowpea.

| **Species names** | **Common names** | **Median total length** | **Median GC count** | **No. of chromosomes** | **Size in bytes** |
| --- | --- | --- | --- | --- | --- |
| *Arachis hypogaea* | Nuts | 2551.68 | 36.3706 | 20+2 | 2.5 GB |
| *Cajanus cajan* | Peas | 620.626 | 33.6052 | 11+2 | 620 MB |
| *Cicer arietinum* | Peas | 510.877 | 32.4428 | 8+2 | 510 MB |
| *Glycine max* | Bean | 997.663 | 35.1918 | 20+3 | 997 MB |
| *Lupinus angustifolius* | Lupins | 557.909 | 33.5664 | 20+1 | 557 MB |
| *Mucuna pruriens* | Pulses | 397.042 | 31.4 | 1WGS | 397 MB |
| *Phaseolus coccineus* | Dry beans | 371.086 | 32.1 | 1WGS | 371 MB |
| *Phaseolus vulgaris* | Dry beans | 535.413 | 36.8761 | 11+1 | 535 MB |
| *Pisum sativum* | Dry peas | 4257.93 | 37.7 | 1WGS | 4.2 GB |
| *Vicia faba* | Dry broad beans | 80.3627 | 38.9 | 1WGS | 80 MB |

Table 15: List of genome composition and distribution of selected leguminous plants for rice bean genome alignment.

| **Species** | **Total seq - LCB aligned** | **Total seq in genome** | **Difference** | **% LCB alginment with rice bean** |
| --- | --- | --- | --- | --- |
| *Vigna radiata* | 333308464 | 310358996 | 22949468 | 93.11465 |
| *Vigna angularis* | 372736198 | 345852835 | 26883363 | 92.78756 |
| *Phaseolus vulgaris* | 521076696 | 512873800 | 8202896 | 88.29 |
| *Cajanus cajan* | 592970700 | 523554643 | 69416057 | 86.56 |
| *Vicia faba* | 80362707 | 64732247 | 15630460 | 80.55011 |
| *Vigna unguiculata* | 473456283 | 378152459 | 95303824 | 79.87062 |
| *Glycine max* | 979046046 | 774075889 | 204970157 | 79.0643 |
| *Ciser arietinum* | 530893862 | 366661949 | 164231913 | 69.06502 |
| *Lupinus angustifolius* | 609203021 | 360776573 | 248426448 | 59.22107 |
| *Phaseolus coccineus* | 371085572 | 93733027 | 277352545 | 25.25914 |
| *Mucuna Pruriens* | 397042285 | 57884047 | 339158238 | 14.57881 |

Table 16: Percentage of complete genome alignment from all selected species having full genome annotation profile.

| **Leguminous plants** | **Postanthesis** | **Photoperiod/day** | **Tons/yr** |
| --- | --- | --- | --- |
| *Arachis hypogaea* | 61 to 105 days | 8 | 3000000 |
| *Cajanus cajan* | 100-120 days | 16 | 370000 |
| *Canavalia ensiformis* | 120 days | 8 | 15300000 |
| *Canavalia gladiata* | 110 - 120 days | 12 | 1310000 |
| *Cicer arietinum* | 145-150 days | 16 | 1150000 |
| *Cyamopsis tetragonoloba* | 120-150 days | 16 | 350000 |
| *Dolichos lablab* | 120 days | 18 | 1900000 |
| *Glycine max* | 45 to 65 days | 10 | 2860000 |
| *Lablab purpureus* | 120 days | 8 | 461734 |
| *Lens culinaris* | 70 - 120 days | 12 | 434000 |
| *Lupinus albus* | 106 to 180 days | 8 | 40000 |
| *Lupinus angustifolius* | 105-150 days | 8 | 120000 |
| *Lupinus luteus* | 165 days | 8 | 120000 |
| *Lupinus mutabilis* | 150 days | 12 | 450000 |
| *Lupinus perennis* | 15-29 days | 12 | 2400000 |
| *Mucuna pruriens* | 140 days | 12 | 130000 |
| *Pachyrhizus erosus* | 150 days | 14 | 400000 |
| *Phaseolus acutifolius* | 60-120 days | 12 | 2400000 |
| *Phaseolus coccineus* | 51 to 60 days | 12 | 850000 |
| *Phaseolus lunatus* | 65 days | 14 | 1900000 |
| *Phaseolus vulgaris* | 50 - 250 days | 9 | 3200000 |
| *Pisum sativum* | 72 days | 12 | 2800000 |
| *Psophocarpus tetragonolobus* | 132 days | 12 | 13000 |
| *Vicia faba* | 80 to 100 days | 8 | 500000 |
| *Vicia sativa* | 44–52 days | 16 | 969000 |
| *Vigna aconitifolia* | 75–90 days | 16 | 100000 |
| *Vigna angularis* | 110 to 120 days | 12 | 700000 |
| *Vigna mungo* | 70 - 75 | 16 | 50000 |
| *Vigna radiata* | 90–120 days | 8 | 104500 |
| *Vigna subterranea* | 120–150 | 12 | 160378 |
| *vigna umbellata* | 120-150 | 12 | 300000 |
| *Vigna unguiculata* | 120 days | 12 | 45000 |

Table 17: Distributions of Postanthesis, photoperiod per day and productive range per year for all selected leguminous plants.

| **Species** | **Sequences** | **% Tot. seq aligned** | **Sequence aligned** | **% of total seq aligned with rice bean** |
| --- | --- | --- | --- | --- |
| *Pachyrhizus erosus* | 39 | 76.92 | 24 | 61.54 |
| *Dolichos lablab* | 1805 | 62.99 | 789 | 43.71 |
| *Lablab purpureus* | 1805 | 62.99 | 789 | 43.71 |
| *Vigna mungo* | 1055 | 75.83 | 391 | 37.06 |
| *Lupinus luteus* | 709 | 69.53 | 242 | 34.13 |
| *Vigna aconitifolia* | 1073 | 71.39 | 315 | 29.36 |
| *Lens culinaris* | 26343 | 30.96 | 7017 | 26.64 |
| *Lupinus albus* | 9864 | 26.20 | 1908 | 19.34 |
| *Cyamopsis tetragonoloba* | 16569 | 24.94 | 3190 | 19.25 |
| *Canavalia ensiformis* | 52 | 34.62 | 10 | 19.23 |
| *Psophocarpus tetragonolobus* | 75 | 24.00 | 11 | 14.67 |
| *Vigna subterranea* | 31 | 22.58 | 3 | 9.68 |
| *Phaseolus lunatus* | 986 | 9.43 | 82 | 8.32 |
| *Canavalia gladiata* | 38 | 13.16 | 3 | 7.89 |
| *Vicia sativa* | 1138 | 17.84 | 67 | 5.89 |
| *Lupinus mutabilis* | 68 | 7.35 | 4 | 5.88 |
| *Lupinus perennis* | 26 | 19.23 | 0 | 0.00 |

Table 18: Percentage distribution of rice bean genes aligned with genes retrieved from actual deposition at NCBI Nucleotide database.

| **Species Notation** | **Species** | **Tot bp blocks aligned** | **Total length of the genome** | **% of tot LCB** | **% with rice bean genome** |
| --- | --- | --- | --- | --- | --- |
| roa | Rauvolfia serpentina | 161099773 | 179746725 | 89.625985 | 43.3 |
| caa | Camptotheca acuminata | 81953592 | 145899039 | 56.171441 | 35.2 |
| dpa | Digitalis purpurea | 126940164 | 127551451 | 99.520753 | 30.8 |
| gba | Ginkgo biloba | 19139196 | 125927755 | 15.198553 | 30.4 |
| rsa | Rosmarinus officinalis | 77676714 | 120161978 | 64.643338 | 29 |
| pqa | Panax quinquefolius | 81232884 | 112315063 | 72.325903 | 27.1 |
| cra | Cannabis sativa | 88845168 | 107796557 | 82.4193 | 26 |
| epa | Echinacea purpurea | 50867288 | 102468088 | 49.642078 | 24.7 |
| csa | Catharanthus roseus | 85738509 | 92280572 | 92.910682 | 22.3 |
| hpa | Hypericum perforatum | 44412301 | 87389108 | 50.821323 | 21.1 |
| dva | Dioscorea villosa | 10296467 | 77321611 | 13.316416 | 18.6 |
| aba | Atropa belladonna | 49133508 | 72725089 | 67.560602 | 17.5 |
| voa | Valeriana officinalis | 55758812 | 62865151 | 88.695901 | 15.2 |
| hga | Hoodia gordonii | 23869178 | 29731667 | 80.282004 | 7.17 |

Table 19: List of 14 vital medicinal plants aligned with rice bean complete assembly.
